# Fluorescent protein tagging of adenoviral proteins pV and pIX reveals ‘late virion accumulation compartment’

**DOI:** 10.1371/journal.ppat.1008588

**Published:** 2020-06-25

**Authors:** Søren Pfitzner, Helga Hofmann-Sieber, Jens B. Bosse, Linda E. Franken, Kay Grünewald, Thomas Dobner

**Affiliations:** 1 Heinrich Pette Institute, Leibniz Institute for Experimental Virology, Hamburg, Germany; 2 Center for Structural Systems Biology, Hamburg, Germany; 3 RESIST Cluster of Excellence, Hannover Medical School, Germany; 4 Universität Hamburg, Institute for Biochemistry and Molecular Biology, Hamburg, Germany; Stony Brook University, UNITED STATES

## Abstract

The human adenovirus type 5 (HAdV5) causes disease of the upper and lower respiratory tract. The early steps of HAdV5 entry up to genome replication in the host nucleus have been extensively studied. However, late stages of infection remain poorly understood. Here, we set out to elucidate the spatiotemporal orchestration of late adenovirus nuclear remodeling in living cells. We generated virus mutants expressing fluorescently tagged protein IX (pIX) and protein V (pV), a capsid and viral genome associated protein, respectively. We found that during progeny virion production both proteins localize to a membrane-less, nuclear compartment, which is highly impermeable such that in immunofluorescence microscopy antibodies can hardly penetrate it. We termed this compartment ‘late virion accumulation compartment’ (LVAC). Correlation between light- and electron microscopy revealed that the LVAC contains paracrystalline arrays of viral capsids that arrange tightly packed within a honeycomb-like organization of viral DNA. Live-cell microscopy as well as FRAP measurements showed that the LVAC is rigid and restricts diffusion of larger molecules, indicating that capsids are trapped inside.

## Author summary

Understanding the regulation of adenovirus morphogenesis is not only of interest to cell biologists but is also key to define novel drug targets as well as to optimize adenoviruses as tools for gene therapy. While early steps of the adenovirus ‘life cycle’ are well understood, it is currently debated how, when and where capsid components associate with viral DNA. Here we used a combination of imaging methods to detail virus-induced spatiotemporal changes at late stages of infection. We found that HAdV5 induces a structured, membrane-less nuclear compartment. In this compartment capsids are closely packed within a honeycomb-like organization of replicated DNA, such that the newly formed particles appear to be trapped and show very little motility. Interestingly, we found a clear discrepancy between immunostaining and fluorescent fusion tagging, indicating a limited penetration of immunostains into this compartment. Since other pathogens induce similar compartments during replication, interpretation of immunostaining data requires careful evaluation.

## Introduction

The human adenovirus type 5 (HAdV5) is a potent pathogen infecting the human respiratory tract while also posing a useful vector system in gene therapy [1,2]. While early events such as entry, endosomal escape and early genome replication have been extensively studied, it is unclear how, when and where capsid constituents and replicated genomes associate in the infected host nucleus. Evidence exists favoring either sequential packaging of the viral genome through a portal protein into the pre-formed capsid or a process of assembly of capsid proteins around the viral genome [3–6].

Importantly, virus assembly is spatially associated with viral replication centers being the source of newly replicated viral genomes. A common indicator for adenovirus replication centers is the viral DNA-binding protein (DBP) [7]. DBP acts as a single-strand DNA binder to stabilize and protect the individual strands and aids in strand unwinding in an ATP-independent mechanism [8,9]. While DBP is associated with replicating DNA, it does not mark replicated genomes. It is therefore unclear when and where capsid constituents associate with ready-to-be packaged genomes during assembly.

Aiming to shine more light onto the spatiotemporal orchestration of nuclear remodeling by adenoviruses, we focused on the two viral proteins pV and pIX as markers for viral genomes and capsids since they have been previously reported to tolerate fusions to fluorescent proteins [10–13]. The minor core protein pV is located within the virus capsid and is exclusively found within the genus of mastadenoviruses [14,15]. Although, no functional motifs have been identified, it is believed to act as a core-capsid bridging protein that interacts strongly and non-specifically with DNA and binds to other core proteins such as pVI and pVII [16–19]. The minor capsid protein pIX has been titled capsid ‘cement’ protein and is located on the outside of the virion [20]. While not essential in virion assembly, deletion mutants of pIX show reduced capsid thermostability [21,22]. pIX has further been implicated in transcriptional regulation and nuclear reorganization but its deletion results in only minor reduction in virus titers [23,24].

The addition of fluorophores to the N- or C-terminus of proteins of interest is easily achieved within plasmid protein expression systems, whereas viral genomes are limited by a space constraint of the packaged genome within the viral capsid. However, the use of fluorescent fusion protein tags in the context of the adenoviral genome was facilitated through engineering of adenoviruses as protein delivery vectors [25]. For that purpose, adenoviral non-essential genes are replaced by non-viral genes of interest. While deletion of early regions E1 and E2 results in replication-deficient virions, it has been shown that substitutions within the adenoviral E3 region are especially suitable for retaining replication capability while for example simultaneously delivering therapeutic proteins such as cytosine deaminase, TNFα or MCP-3 [26,27]. Analogously, viral protein labelling can be achieved through either adding a second gene copy together with the fusion tag within the E3 region or by modifying the native gene in its natural genetic background. We employed the latter method to achieve fluorescent tagging of pV and pIX.

In this study, we use a combination of live-cell fluorescence microscopy and correlated electron microscopy techniques to follow the spatiotemporal coordination of capsid-genome interactions at the subcellular level. Our results revealed the formation of a to our knowledge uncharacterized intranuclear viral compartment at late stages of infection. Importantly, this compartment was not fully characterizable with immunofluorescence staining. We have observed the compartment to be a protein phase, which may prevent the penetration of the immunofluorescence stain. As we have found arrays of fully formed virions contained within, we postulate the assembly and accumulation of viral particles in this compartment which could be aided by the concentration of all necessary viral factors. Therefore, we label it ‘late virion accumulation compartment’ (LVAC).

## Results

### Fluorescent protein tags for detection of viral proteins in live-cell fluorescence microscopy

**Fig 1:**
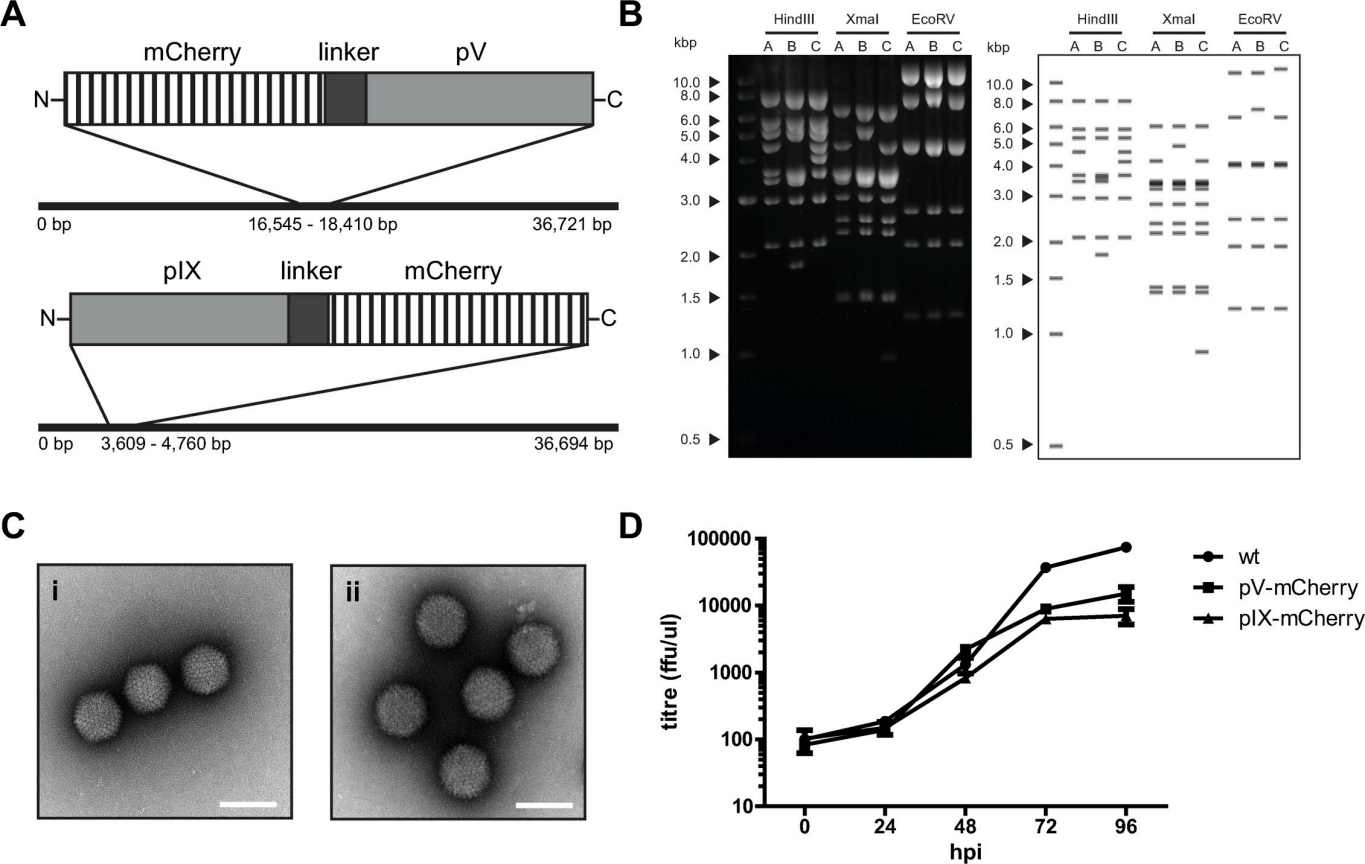
HAdV5 tolerates fusion of proteins pV and pIX to mCherry. (A) Overview of mCherry-fusion constructs. Two HAdV5 mutants were generated. Protein V and protein IX were fused to the mCherry fluorophore in combination with a varying flexible linker region. mCherry was fused N-terminally to pV and C-terminally to pIX without disrupting adenoviral splice sites. (B) Restriction analysis of wt, pV-mCherry and pIX-mCherry bacmid DNA (lanes labelled A, B, C) including *in-silico*-prediction of restriction pattern. (C) negative stain TEM of purified virus particles. The virus particles of each mutant were purified, contrasted with uranyl acetate negative stain and imaged by transmission electron microscopy. Scalebars indicate 100 nm. HAdV5 pV-mCherry particles are shown in i) HAdV5 pIX-mCherry particles are shown in ii). (D) Growth curve of virus particle release from infected A549 cells from 0 to 96 hpi.

HAdV5 pV and pIX proteins were fused to the monomeric fluorophore mCherry. As the localization of both proteins was to be studied in infection rather than transfection experiments, the pV and pIX genes were modified within the HAdV5 genome. The fluorescent mutants of HAdV5 were generated by recombineering in *E*. *coli* (Fig 1A) [28]. Virus particles were produced after transfection of the linearized viral genome into H1299 cells. The integrity of the modified viral genome was checked via restriction enzyme analysis and the appearance of purified virus particles was evaluated by negative stain transmission electron microscopy (TEM) (Fig 1B and 1C). Both HAdV5 pV-mCherry and HAdV5 pIX-mCherry genomes show the *in-silico* predicted restriction pattern and the virus particles showed a typical icosahedral shape indicating normal virion assembly. The release of mutant virus particles from infected cells between 24 and 48 hours post infection (hpi) was comparable to wild type (wt) infection. Only from 72 to 96 hpi 10-fold more wt virus was released (Fig 1D).

### Fluorescent fusion proteins pV-mCherry and pIX-mCherry reveal a large intranuclear viral protein compartment

**Fig 2:**
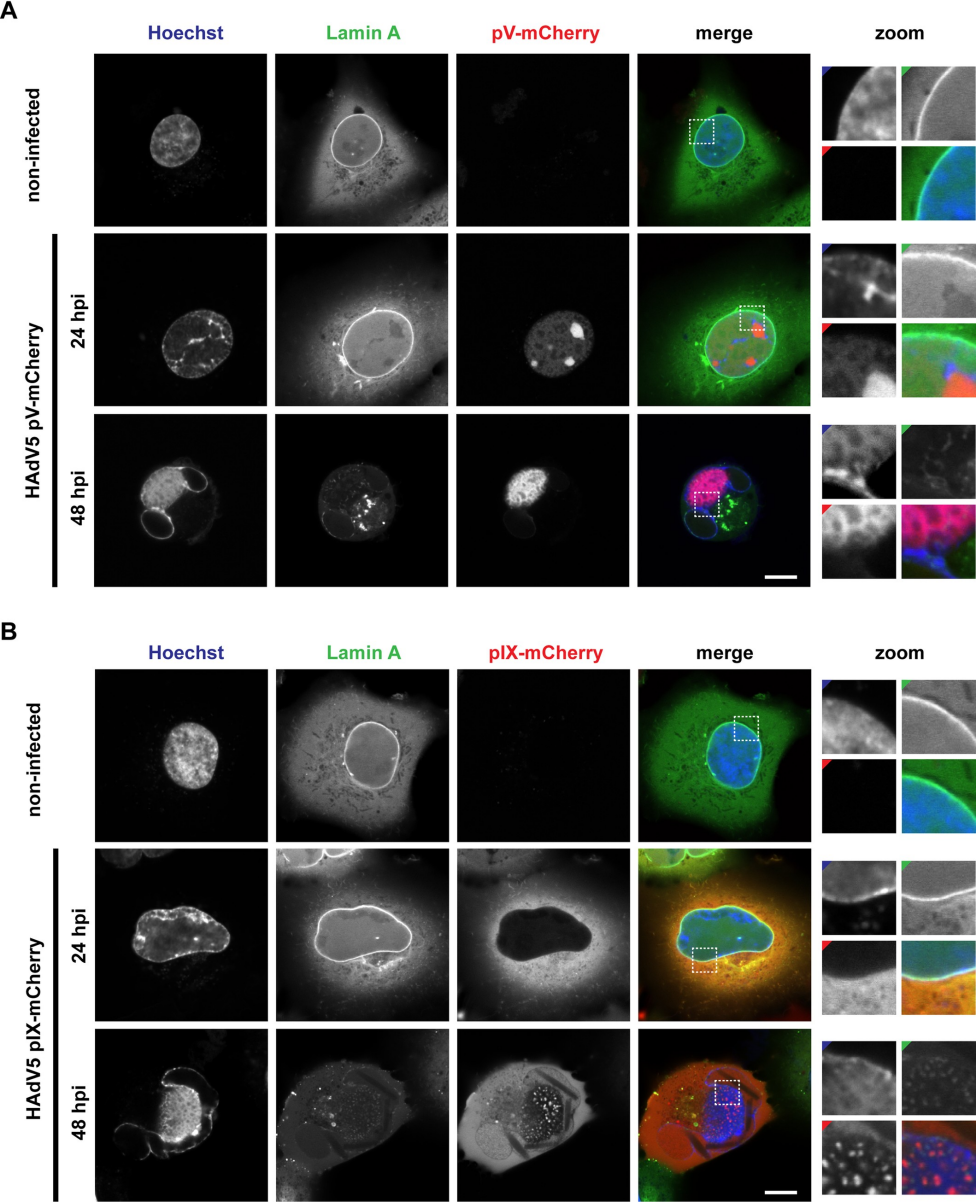
pV-mCherry and pIX-mCherry display a large intranuclear compartment late in infection. (A) Infection of A549 cells with HAdV5 pV-mCherry at 24 hpi and 48 hpi. (B) Infection of A549 cells with HAdV5 pIX-mCherry at 24 hpi and 48 hpi. The cells were imaged by live-cell confocal laser-scanning fluorescence microscopy. A representative cell is shown for each condition. The dsDNA signal is represented by Hoechst 33342 stain (Hoechst). The nuclear lamina is represented by a GFP-nanobody recognizing lamin A (Lamin A). pV and pIX localization is detected through the viral pV-mCherry and pIX-mCherry fusion construct (pV-mCherry/pIX-mCherry). The signal overlap is represented in color (merge). Nuclear regions of interest are enlarged (zoom) with colored corners indicating the channel color. Scalebars indicate 10 μm.

The localization of pV-mCherry and pIX-mCherry in HAdV5 mutant infection was analyzed via laser-scanning confocal fluorescence microscopy. To be able to put pV and pIX signals into context within infected cells we applied co-stains targeting the nuclear envelope and DNA. The nuclear envelope was visualized via a stably expressed anti-lamin A nanobody-GFP fusion protein. Double-stranded DNA was stained with Hoechst 33342. The infection phenotype of cells infected with either fluorescent virus mutant was analyzed at 24 and 48 hpi (Fig 2).

In non-infected cells, Hoechst 33342 stained the entire nucleus and the lamin A nanobody localized to the intact nuclear envelope. The nanobody gave a moderate background staining in the cytoplasm, that is not detected with a lamin A antibody (S1 Fig). In infected cells, both DNA and lamin A localization changed considerably between 24 and 48 hpi. Hoechst 33342 stained a DNA-filled compartment in the center of the nucleus as well as marginalized DNA at the nuclear envelope. The lamin A nanobody signal lost most its association with the nuclear envelope and was found to be weakly distributed within the central compartment with only residual signal staining the nuclear envelope.

At 24 hpi, pV-mCherry was weakly expressed throughout the nucleus and enriched in areas, previously identified as the nucleoli [18]. Here, the protein was noticeably excluded from ring- and dot-like structures resembling DBP replication centers [7]. At this timepoint, pIX-mCherry was mainly expressed in the cytoplasm and only a very weak signal was recorded in the nucleus. The nuclear lamina was still intact in cells infected with either fluorescent mutant as the lamin A nanobody clearly located to the nuclear envelope. The DNA signal was already undergoing reorganization, as DNA marginalization to the nuclear envelope could be seen.

At 48 hpi, pV-mCherry protein was localized as a single large compartment within the nucleus and a few individual foci close to the nuclear envelope. pIX-mCherry was observed in the cytoplasm as well as the nucleus. In addition to homogenously distributed signal, pIX-mCherry formed individual spots inside the separated nuclear compartment indicated by Hoechst 33342. Such pIX-mCherry spots were observed in 77% of infected cells observed in a single confocal plane, indicating a common occurrence in infection. The large compartment of pV-mCherry, pIX-mCherry spots and DNA will be referred to as late virion accumulation compartment (LVAC).

As control for cell line-specific artifacts, the virus mutants were used to infect the lung epithelial cell line H1299 and the lung fibroblast cells line MRC-5. The phenotypes observed for 24 hpi and 48 hpi were comparable to those of A549 cells (S2 and S3 Figs).

### Immunofluorescence staining indicates that LVACs show permeation restrictive properties

**Fig 3:**
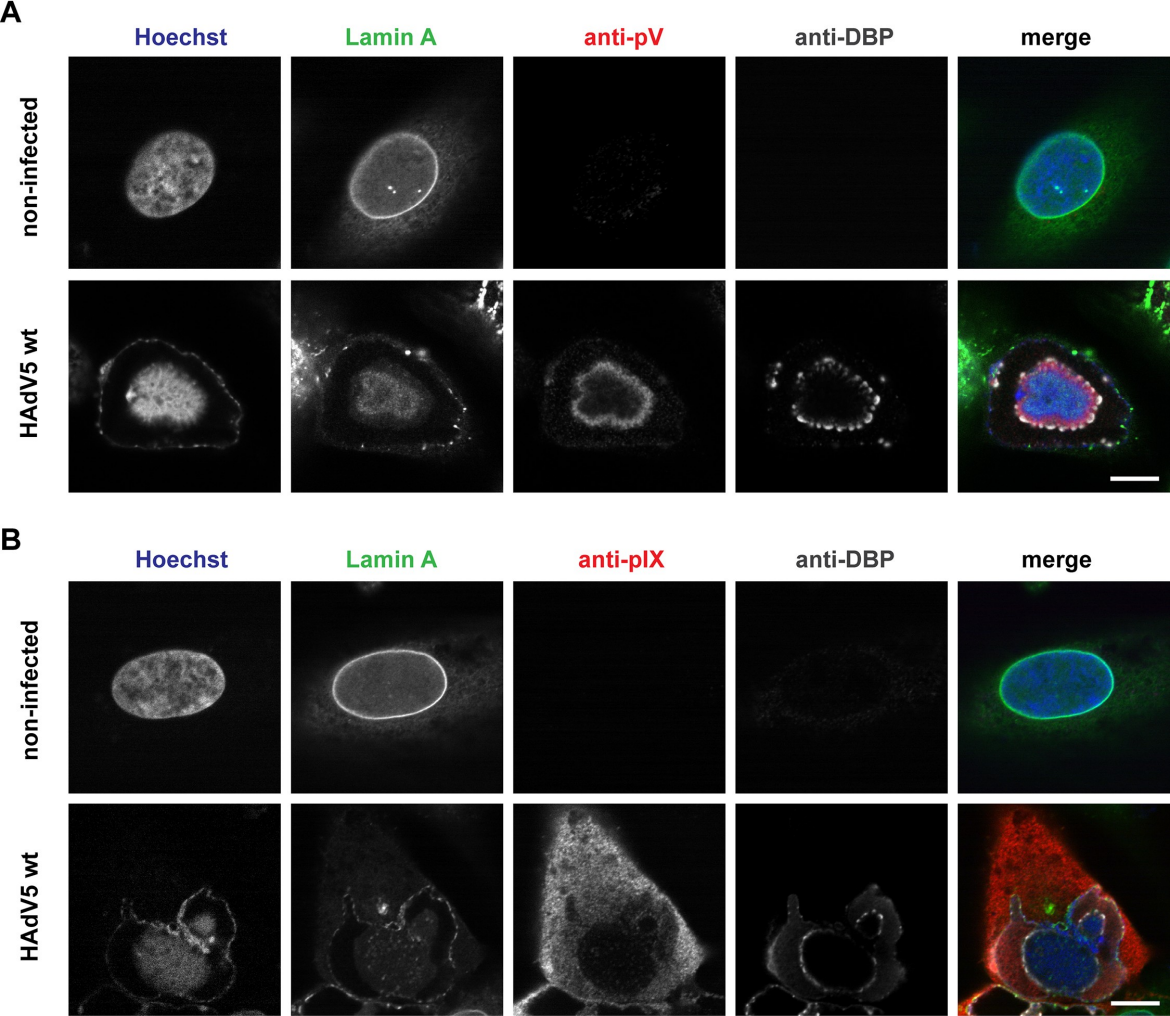
Antibodies against pV and pIX do not fully stain the LVAC. (A) Immunofluorescence labeling of pV in HAdV5 wt infection. (B) Immunofluorescence labelling of pIX in HAdV5 wt infection. A549 cells were infected with HAdV5 wt, fixed at 48 hpi and imaged by confocal laser-scanning fluorescence microscopy. Cells were stained with Hoechst 33342 (Hoechst), and immunostained against pV (anti-pV) or pIX (anti-pIX) and DBP (anti-DBP). The nuclear lamina is represented by a GFP-nanobody recognizing lamin A (Lamin A). The signal overlap is represented in color (merge). A representative non-infected and infected cell is shown for each stain. Scalebars indicate 10 μm.

As control for our fluorescent mutants we performed immunofluorescence staining against pV and pIX aiming to study the LVAC. A549 cells were infected with HAdV5 wt and the infection phenotype was analyzed at 48 hpi. The nuclear envelope was visualized via a stably expressed anti-lamin A nanobody-GFP fusion protein. Double-stranded DNA was stained with Hoechst 33342. Additionally, we stained for DNA-binding protein (DBP) to reveal the involvement of the LVAC with adenovirus replication centers (Fig 3).

As in the live-cell microscopy of the pV and pIX fusion proteins, Hoechst 33342 stained a DNA-filled compartment within the nucleus, which we termed LVAC. However, the antibody stain of pV and pIX differed from the phenotype of the fusion proteins. The pV antibody localized in a ring-like structure surrounding the LVAC. Noticeably, the pV antibody signal gradually decreased towards the center of the compartment. The DBP antibody also stained a ring-like structure which consisted of individual DBP foci. As pV, the DBP ring was located around the LVAC and the antibody signal decreased towards the center. The pIX antibody signal was mainly located in the cytoplasm of cells. Instead of pIX spots, which were visible in the fusion proteins, the area of the LVAC was not stained by the pIX antibody. Since the same DNA compartment was visible in HAdV5 wt infection but none of the antibodies against pV, pIX and DBP showed signal in the center of the nucleus, we hypothesized a limited antibody penetration into the LVAC.

### Colocalization analysis reveals lack of antibody penetration only in late infection

**Fig 4:**
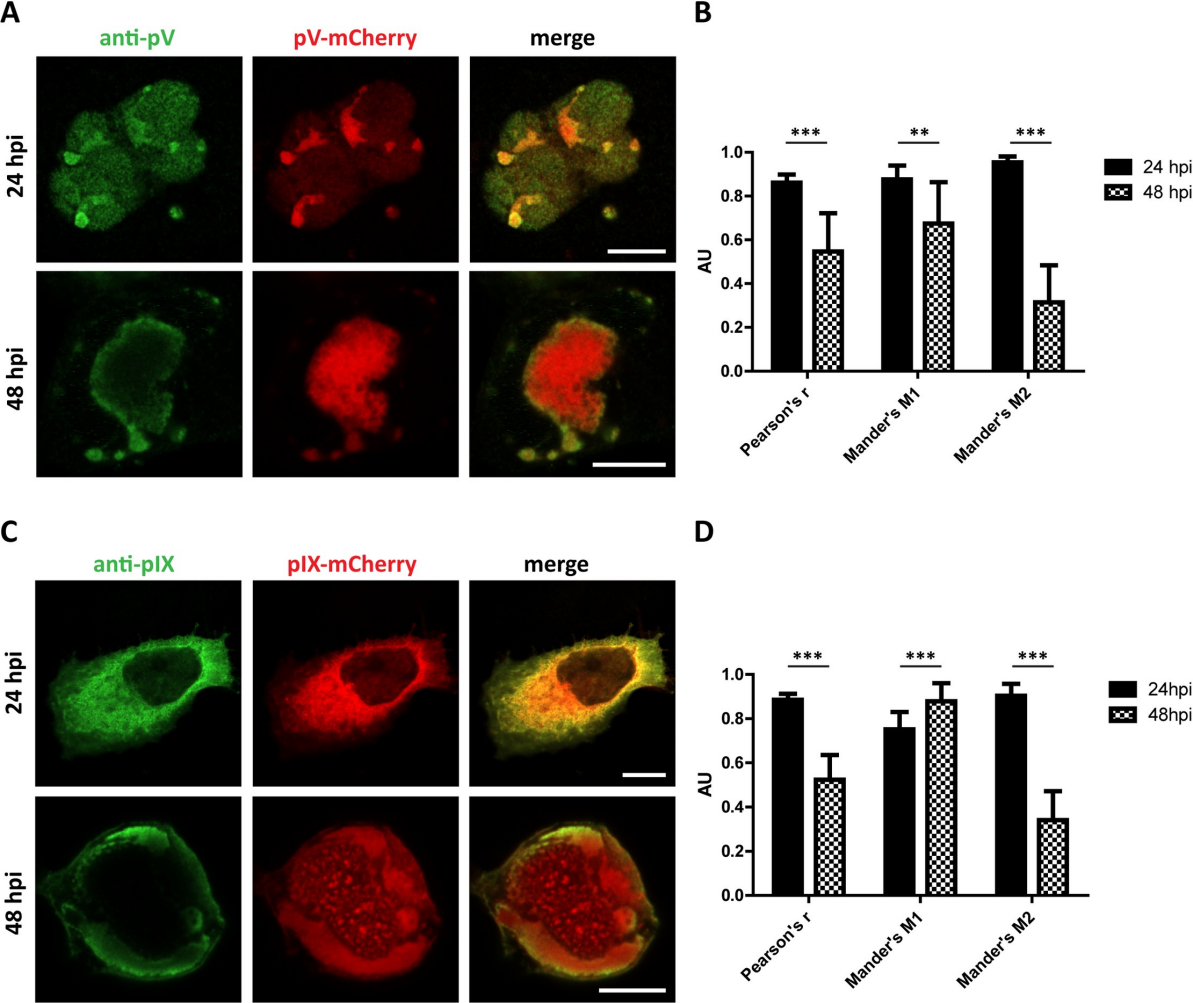
The signal of mCherry fusion constructs and pV and pIX antibody only diverges in late infection. (A/C) Immunofluorescence stain of pV-mCherry/pIX-mCherry with anti-pV/pIX antibody. A549 cells were infected with HAdV5 pV-mCherry and HAdV5 pIX-mCherry. At 24 and 48 hpi the cells were fixed and immunostained with pV or pIX antibodies and imaged in confocal fluorescence microscopy. A representative cell for both time points of HAdV5 pV-mCherry and HAdV5 pIX-mCherry infection is shown. Scalebars indicate 10 μm. (B/D) Quantification of mCherry-antibody signal colocalization as represented by the average Pearson’s coefficient r and Mander’s coefficients M1 and M2 (n = 12). Statistical significance was calculated using Student’s t-test with preceding F-test and is indicated as stars above the bars.

To statistically analyze and confirm that the fusion protein mCherry was indeed labelling pV and pIX, the colocalization of tagged pV and pIX and the corresponding antibody stain was tested. A549 cells were infected with HAdV5 pV-mCherry or HAdV5 pIX-mCherry, fixed at 24 hpi or 48 hpi and stained with pV and pIX antibodies (Fig 4).

At 24 hpi, the antibody stains colocalized strongly with the respective fusion proteins. Mander’s coefficients were measured to describe the overlap of antibody signal with mCherry signal (M1) and mCherry signal with antibody signal (M2). The Pearson’s correlation coefficient was measured to quantify the overall pixel intensity correlation. pV measured Mander’s coefficients M1 and M2 of 0.88 and 0.95 as well as a Pearson’s coefficient of 0.86. pIX measured Mander’s coefficients M1 and M2 of 0.75 and 0.90 as well as a Pearson’s coefficient of 0.89. These values indicate significant colocalization and a strong positive correlation between both signals.

At 48 hpi, the information provided by pV and pIX antibody was heavily reduced since colocalization between antibody signal and fusion protein signals diverged. pV antibody signal still colocalized strongly with the pV-mCherry signal, as shown by a Mander’s coefficient M1 of 0.67. However, pV-mCherry signal colocalized only partially with the pV antibody signal, as shown by a Mander’s coefficient M2 of 0.31. The same phenomenon was observed for pIX, measuring Mander’s coefficients M1 of 0.88 and M2 of 0.34. These results showed that our mCherry fusion tags achieved comparable stains of pV and pIX to the antibodies staining at early time points. Only at late stages of infection the staining capability of both probes diverged. Here, the antibodies only detected a spatially more accessible subpopulation of pV and pIX. The fusion tags provided more detail and were able to uncover structures that were not detectable with the antibodies.

As additional controls, the above-mentioned co-stains were also repeated in the context of staining DBP and DNA at 48 hpi (S4 Fig). Both DBP and Hoechst 33342 signals did not differ phenotypically from wt infection, indicating that the infection phenotype observed in the HAdV5 mutants is comparable to HAdV5 wt.

### Live-cell imaging reveals congregation of pV-mCherry rings to form the LVAC

**Fig 5:**
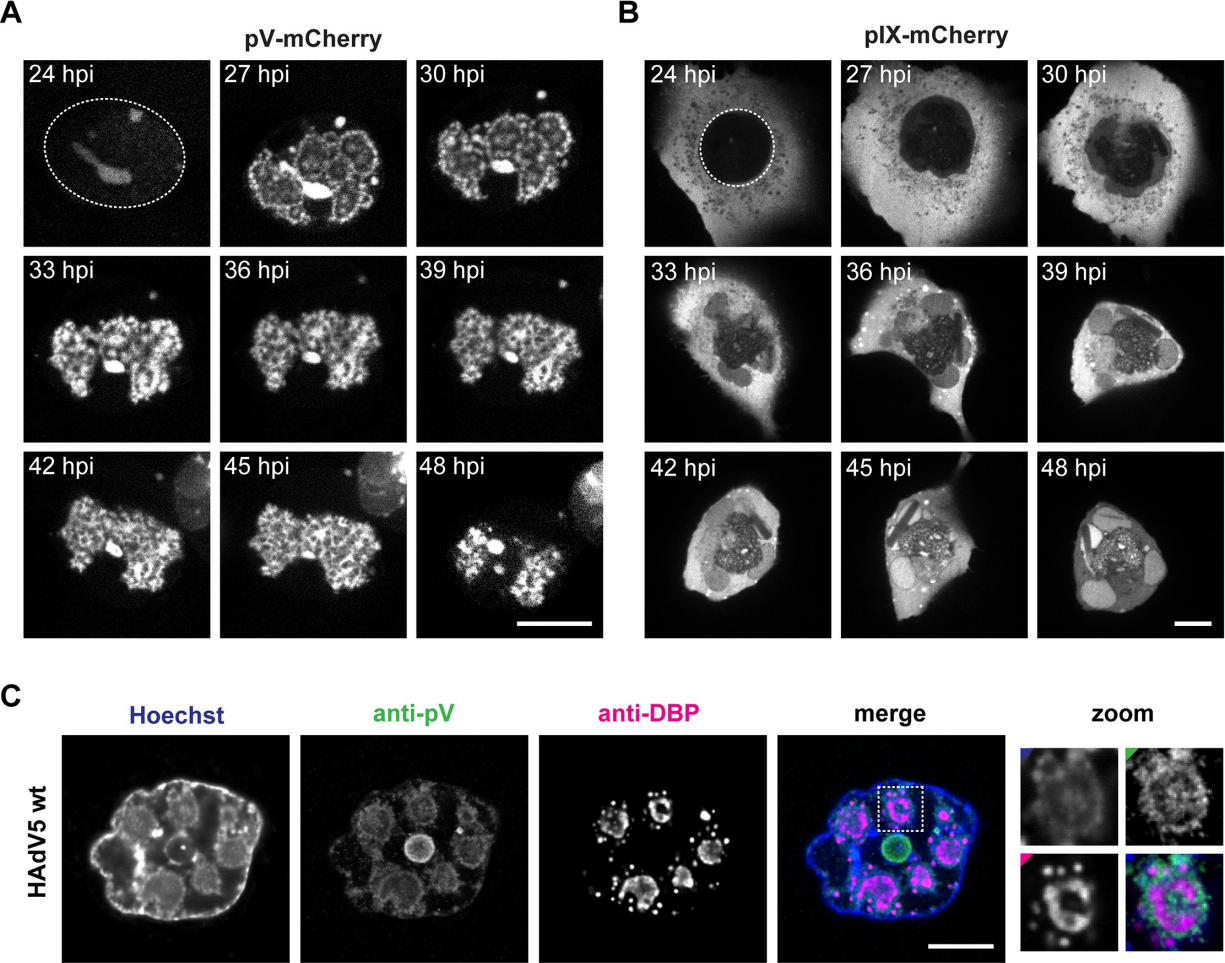
pV forms ring-like structures within the nucleus before congregating into the LVAC. (A) Infection progression of HAdV5 pV-mCherry infected cell. (B) Infection progression of HAdV5 pIX-mCherry infected cell. The cells were selected for imaging in live-cell spinning-disk confocal fluorescence microscopy at 24 hpi. The initial nuclear boundaries for the HAdV5 pV-mCherry or pIX-mCherry infected cell are visualized as a dashed line. The cells were imaged as z-stack to be able to adjust for z-movement of infected cells. The image histograms along infection progression have been modified to adjust for changes in protein intensity. (C) Co-stain of pV showing the ring-like phenotype and DBP replication centers in HAdV5 wt infection. A549 cells were infected with HAdV5 wt, fixed at 24 hpi, stained with Hoechst 33342 (Hoechst), and immunostained against pV (anti-pV) and DBP (anti-DBP). Cells were imaged by confocal laser-scanning fluorescence microscopy. The signal overlap is represented in color (merge). A DBP replication ring is enlarged (zoom) with colored corners indicating the channel color. Scalebars indicate 10 μm.

Next, the infection progression of both virus mutants was followed by time-lapse confocal spinning-disk fluorescence microscopy. Cells were selected at 24 hpi and imaged in 1 h intervals until 48 hpi. Through this method, cellular morphological changes were observable, that connected the phenotypes at 24 and 48 hpi (Fig 5A and 5B).

During the HAdV5 pV-mCherry infection, the initially weak and uniformly spread pV-mCherry signal condensed to ring-like structures within the first hour of imaging before further congregating to the single LVAC that can be observed at 48 hpi. These intermediate rings resembled the ring structures observed for DNA-binding protein (DBP) replication centers in adenovirus infection [7]. During the entire HAdV5 pIX-mCherry infection pIX-mCherry signal was detected in the cytoplasm. Initially, only a weak pIX-mCherry signal was detected in the nucleus. The signal intensity increased continuously throughout progression of the infection. The appearance of pIX-mCherry spots can be observed between 30 and 33 hpi.

To investigate the formation of pV-mCherry ring-like structures in relation to DBP replication centers, we further analyzed the intermediate pV phenotype with immunofluorescence confocal microscopy. A549 cells were infected with HAdV5 wt and fixed at 24 hpi. The viral proteins pV and DBP were indirectly stained with antibodies and dsDNA was stained with Hoechst 33342. Cells showing the phenotype of pV rings were analyzed (Fig 5C). In these cells, DBP was localized as ring-like structures and smaller foci within the nucleus. Interestingly, both Hoechst 33342 signal and pV protein were localized around the DBP replication centers and in the center of DBP replication rings, indicating that pV-mCherry ring formation is linked to the localization of DBP replication centers in infected cells.

### The LVAC substructure resembles a honeycomb-like distribution of pV-mCherry and dsDNA surrounding pIX-mCherry spots

**Fig 6:**
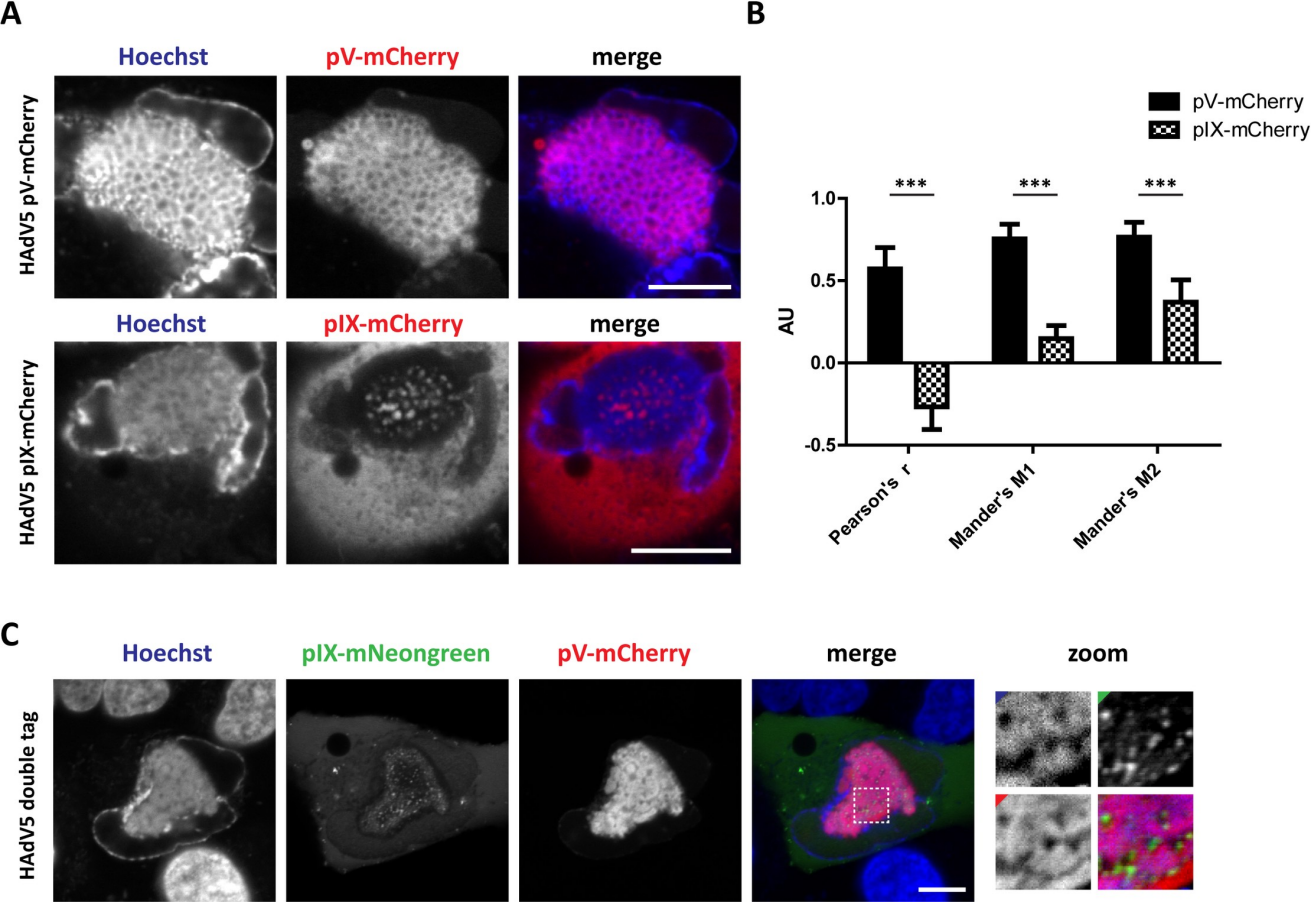
Inside the LVAC, pIX-spots are located within cavities of a dsDNA and pV honeycomb-like organization. (A) Colocalization of pV-mCherry and pIX-mCherry and dsDNA in the LVAC. A549 cells were infected with HAdV5 pV-mCherry and HAdV5 pIX-mCherry. At 48 hpi, the cells were stained with Hoechst 33342 and imaged in confocal laser-scanning live-cell fluorescence microscopy. A representative cell for each virus mutant is shown. (B) Colocalization quantification between Hoechst 33342 and pV-mCherry or pIX-mCherry signal in the LVAC. The overlap between both signals is represented by the average Pearson’s coefficient r and Mander’s coefficients M1 and M2 (n = 12). Statistical significance was calculated using Student’s t-test with preceding F-test and is indicated as stars above the bars. C) Colocalization of pV-mCherry and pIX-mNeongreen in the LVAC. A549 cells were infected with the double mutant HAdV5 pV-mCherry pIX-mNeongreen and imaged at 48 hpi. A region of the LVAC is enlarged (zoom) showing pIX-mNeongreen spots to populate the cavities of the pV-mCherry honeycomb. Colored corners indicate the channel color. Scalebars indicate 10 μm.

The LVAC containing dsDNA, pV and spots of pIX was further investigated. At closer inspection, pV-mCherry protein distribution was observed to resemble a honeycomb-like organization of a connected network of protein with empty volumes in between. This honeycomb distribution was also found for dsDNA as indicated by Hoechst 33342 staining. The colocalization between pV-mCherry/pIX-mCherry and Hoechst 33342 signals was quantified (Fig 6).

pV-mCherry and dsDNA significantly colocalized within the LVAC as shown by Mander’s coefficients M1 and M2 of 0.75 and 0.76 as well as a positive Pearson’s coefficient r of 0.57. Cells infected with HAdV5 pIX-mCherry also displayed a dsDNA honeycomb-like organization. In contrast to pV-mCherry, pIX-mCherry spots were located within the cavities of the Hoechst 33342 honeycomb. Mander’s coefficients M1 and M2 of 0.15 and 0.37 were measured, indicating a limited colocalization of both signals. Interestingly, a Pearson’s coefficient r of -0.27 showed a negative correlation between the dsDNA and pIX-mCherry spots indicating pIX-mCherry signal to mainly populate the spaces devoid of dsDNA. These results indicated a significant spatial overlap between dsDNA and pV-mCherry but a spatial separation between dsDNA and pIX-mCherry.

Since a co-stain of pV and pIX was neither achievable with the single fluorescently tagged virus mutants nor with antibody co-labelling, a double mutant of HAdV5 pV-mCherry and pIX-mNeongreen was generated. Although this mutant showed reduced growth, live cell imaging at 48 hpi confirmed the localization of pIX spots within the cavities in the pV honeycomb (Fig 6C).

### Correlation of fluorescence and transmission electron microscopy reveals presence of viral paracrystalline arrays in LVAC

**Fig 7:**
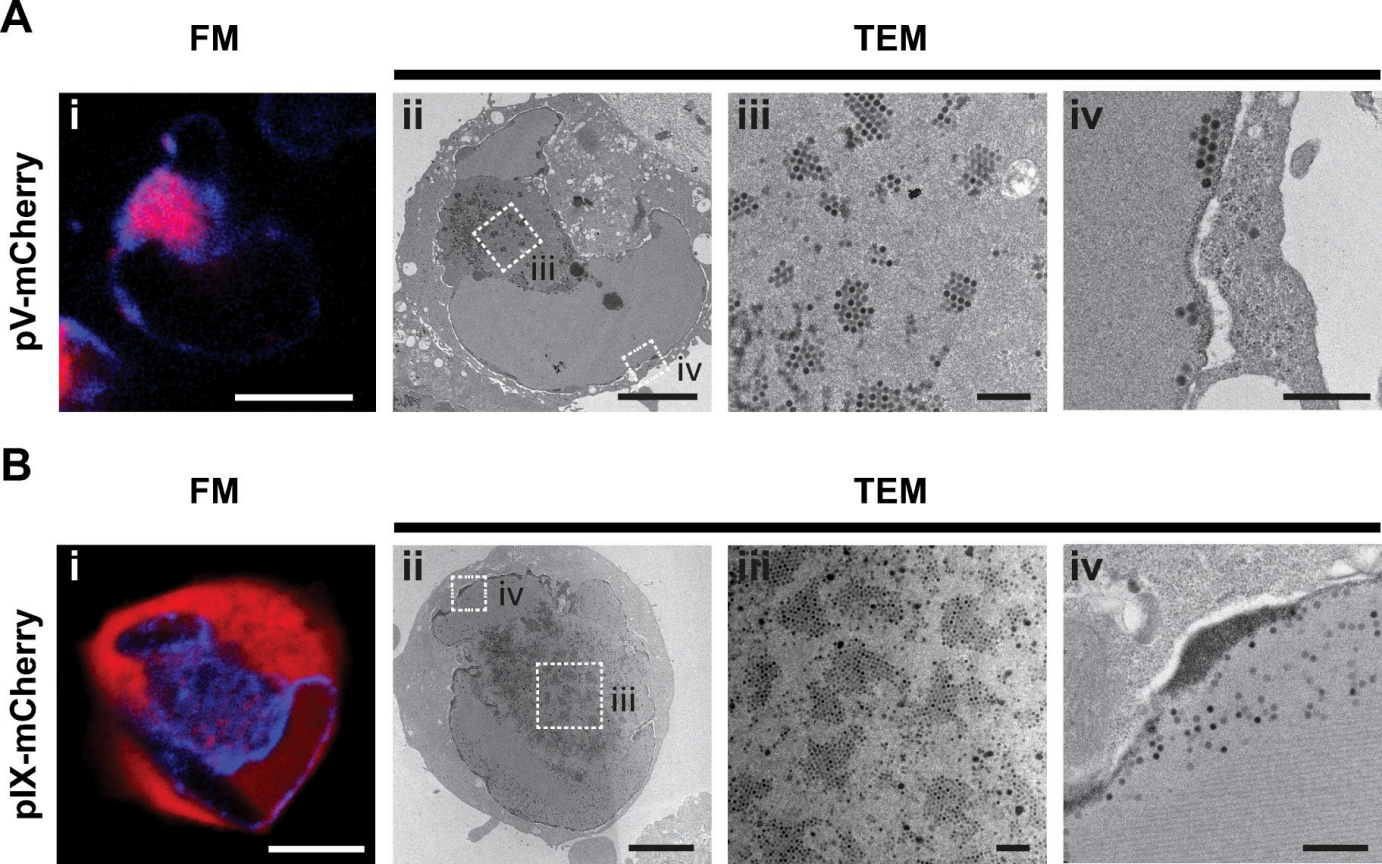
Paracrystalline virus arrays accumulate within the LVAC and correspond to the pIX-mCherry spots found in the LVAC. (A) A549 cells infected with HAdV5 pV-mCherry. (B) A549 cells infected with HAdV5 pIX-mCherry. The cells were stained with Hoechst 33342 and were imaged by live-cell spinning-disk confocal fluorescence microscopy at 48 hpi (FM). Immediately after imaging, the cells were fixed and prepared for transmission electron microscopy (TEM). 50 nm sections of the epon-embedded cells were imaged with TEM. FM cell overviews are shown in images i) with Hoechst 33342 in blue and pV-mCherry and pIX-mCherry in red. TEM cell overviews are shown in images ii) including scalebars indicating 5 μm. Higher magnification images of selected areas of cells are shown in images iii) and iv) for both sections A) and B) including scalebars indicating 0.5 μm. Scalebars for fluorescence images indicate 10 μm.

Thus far, fluorescent tagging of pV and pIX provided information on the subcellular localization of both proteins. To elucidate the identity of pIX-mCherry spots within the LVAC, fluorescence microscopy (FM) was combined with transmission electron microscopy (TEM). First, A549 cells were infected with HAdV5 pV-mCherry and pIX-mCherry. Selected cells were imaged at 48 hpi in live-cell confocal spinning-disk fluorescence microscopy (Fig 7). Immediately after imaging the cells were chemically fixed with glutaraldehyde and subsequently embedded in EPON resin for TEM sectioning into ultrathin slices. The exact same cells were identified and selected for TEM imaging. Comparing FM and TEM images showed that the overall cell shape changed slightly between the two techniques, as the cell retained plasticity in the time period between imaging and fixation. Nonetheless, cellular features such as the position of large protein crystals that appeared as dark shadows in the mCherry channel could be found back in the TEM sections. While the exact ultrastructure of the nuclear envelope is not identical between both images, the overall nuclear kidney shape stayed the same. The LVAC in HAdV5 pV-mCherry and HAdV5 pIX-mCherry infection can be detected by a difference in protein composition in the nucleoplasm resulting in a darker TEM stain. In pV-mCherry infected cells, fully formed capsids could be found within the central compartment (Fig 7Aiii). Interestingly, most virions were clustered in specific regular paracrystalline virus arrays of varying size and shape. Outside the LVAC, virions were detected at the nuclear envelope (Fig 7Aiv). Paracrystalline arrays were also detected in pIX-mCherry infected cells (Fig 7Biii). Comparison between the fluorescence microscopy images of pIX-mCherry positioned the virus arrays within the LVAC. These data suggest that the pIX-mCherry spots result from a local concentration of pIX-mCherry protein, incorporated within fully formed virus capsids. These virus arrays were only found within the LVAC. Outside, virions were detected as single particles (Fig 7Biv).

### Paracrystalline virus arrays are highly immobile within the LVAC

**Fig 8:**
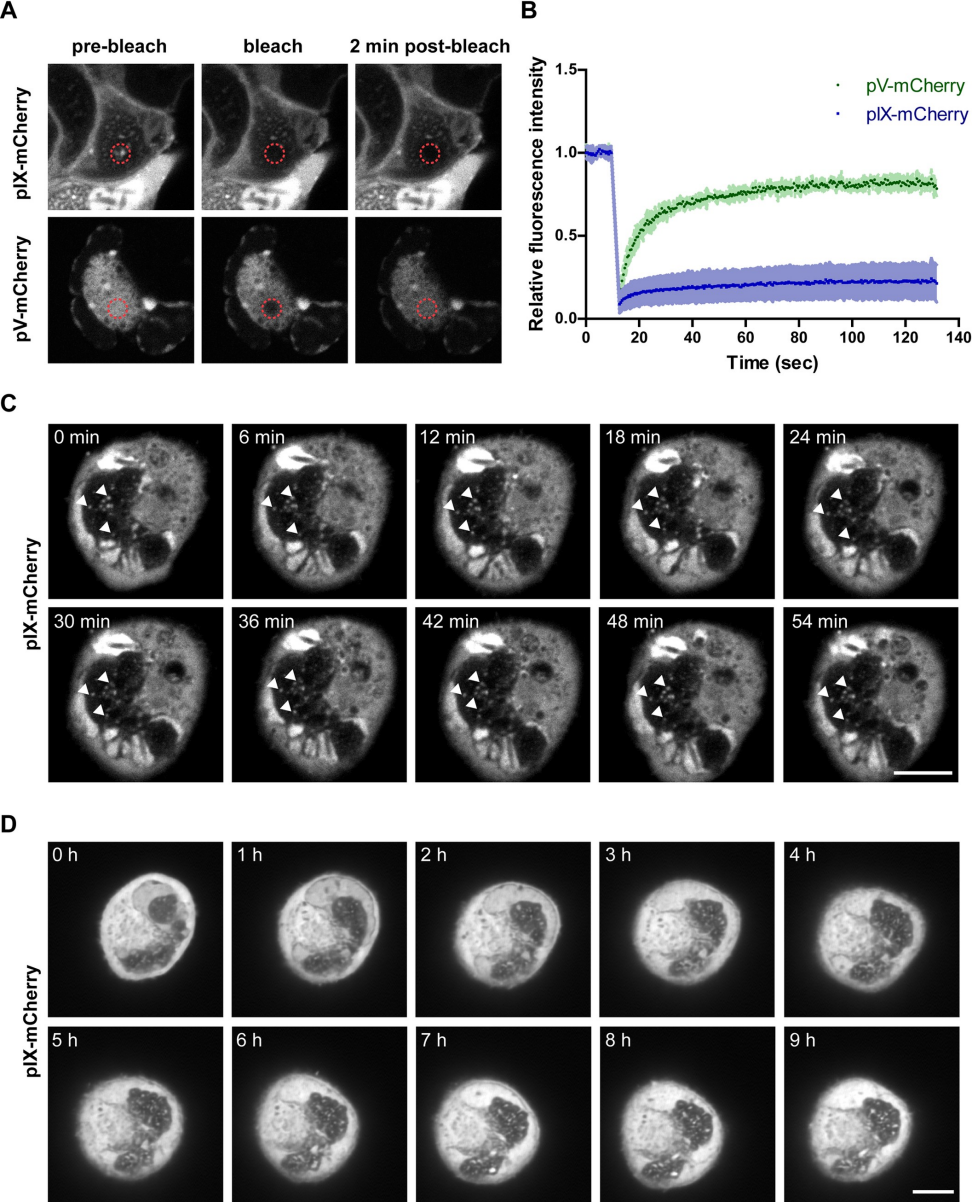
The paracrystalline arrays are highly immobile within LVAC. (A) FRAP laser bleaching of pV-mCherry and pIX-mCherry signal within LVAC constituents in infected A549 cells. A dashed red circle is indicating the bleach area. The cells are shown before bleaching (pre-bleach), at the timepoint of bleaching (bleach) and after 2 min recovery (2 min post-bleach). (B) FRAP quantification. The relative fluorescence intensity change was plotted against time. The average signal change is indicated (n = 6) with the standard deviation indicated as colored underlying area. (C/D) Time-lapse live-cell fluorescence microscopy analysis of A549 cells infected with pIX-mCherry. The position of paracrystalline virus arrays (pIX-mCherry spots) remained largely unchanged over 54 min (indicated by white arrows) and only slowly shifted in the course of multiple hours. Images in D) were denoised using the Noise2Void algorithm [29]. Scalebars indicate 10 μm.

To characterize the motility of the adenoviral proteins pV and pIX located within the LVAC, infected cells were subjected to fluorescence recovery after photobleaching (FRAP) experiments. A short and focused laser pulse aimed at a region of interest (ROI) causes bleaching of the fluorophores located within the ROI. Here, pIX-mCherry spots and pV-mCherry signal in the LVAC were chosen for FRAP analysis. The change in fluorescence signal during a recovery time of 2 min was measured and compared to the background and a non-bleached ROI. The relative fluorescence intensity change over time can be interpreted and provides information about the mobile fraction of the protein of interest (Fig 8). Recovery of pIX-mCherry signal after bleaching was shown to be limited. Through curve fitting the mobile fraction was calculated to be 22% indicating a large fraction of protein to be immobilized in pIX spots within the compartment. Analysis of pV-mCherry showed a different behavior of the protein. Most of the protein signal recovered, with a mobile fraction of 80% indicating only a small fraction of protein to be immobilized within the honeycomb-like organization. Interestingly, despite the different mobile fractions, the half times of recovery were comparable for both proteins at 20 sec for pIX-mCherry and 18 sec for pV-mCherry.

To further analyze the mobility of entire paracrystalline virus arrays within the infected cell, cells showing pIX-mCherry spots in confocal fluorescence microscopy were imaged over 54 min or 9 h. Within the shorter time period of 54 min the position of pIX-mCherry spots within the LVAC remained unchanged (Fig 8C). Only over a longer time period of multiple hours the pIX-mCherry spots shifted position (Fig 8D). This finding together with the FRAP data showed that pIX-mCherry spots, which correspond to paracrystalline virus arrays, are highly immobile.

## Discussion

Most studies on the adenovirus life-cycle focus on early events, including virus-cell attachment within the first minutes of infection, virus endosomal escape within the first hour, the viral genome injection into the nucleus after about 3 hours, and the formation of replication centers from 8 h to up to 24 h post infection [30–33]. Here, we focused on even later timepoints from 24 to 48 hpi and analyzed infection phenotypes through fluorescence and electron microscopy. To study genome-capsid localization in live-cell microscopy we generated virus mutants expressing fluorescently tagged viral proteins pV and pIX. Our results showed nuclear morphological changes of chromatin organization as well as viral proteins DBP, pV and pIX in the late stages of adenoviral infection. We focused in particular on the observation of a large intranuclear compartment of enriched with pV-mCherry and dsDNA surrounding paracrystalline arrays of virions. We termed this compartment the ‘late virion accumulation compartment’ (LVAC).

### Fluorescent fusion proteins can overcome the antibody penetration limitations and visualize the LVAC

We exploited the benefit of fusion protein labelling to analyze morphological changes within individual cells in live-cell microscopy without being reliant on chemical fixation of a population of different cells at various time points. The choice of fusion proteins is often constrained by the functional and structural deficits imposed on the protein through its fusion partner. Adenoviral proteins pV and pIX have been shown to accommodate N-terminal or C-terminal modifications, respectively. Deletion of the C-terminal coiled-coil domain of pIX resulted in fully formed and thermostable capsids and addition of a Flag tag and heparan sulfate binding motif allowed the use of adenovirus capsid for ligand display [10,11]. Similarly, pV fused to EGFP in transfection localizes in an identical manner to the native pV [13,18]. A few studies exist that use pV or pIX fluorescent fusion tags [34–37]. A pV-GFP fusion construct was used to analyze the disassembly of capsids at the nuclear envelope accompanied by loss of pV [38]. pIX-EGFP or pIX-mRFP1 fusion constructs were used to analyze localization of fully formed virions during entry in single cells and mouse tissue and were even combined in a simultaneous labelling approach to track virus particles upon infection. [34–37].

In our control immunofluorescence stainings we observed a lack of antibody penetration into the LVAC, which we were able to overcome with the fluorescent fusion protein mutants. Only at 24 hpi, but not 48 hpi, the pV and pIX antibodies were colocalizing strongly with tagged pV-mCherry and pIX-mCherry (Fig 4). Importantly, this experiment shows that staining impairment did not result from a loss in antibody specificity since we observe no staining impairment at early time points. Additionally, the introduction of mCherry as a fusion partner did not induce the LVAC formation as a phenotypical artifact. The LVAC can be detected in HAdV5 wt infection as well as HAdV5 pIX-mCherry and pV-mCherry infection, as indicated by the formation of a DNA compartment in the center of the nucleus (Figs 2 and 3). A lack of antibody penetration can explain why we only detected pV ring structures on the edges of LVACs in HAdV5 wt infection. Presumably, the congregation of viral proteins led to a phase, which did not allow free permeation of large antibodies. High local amounts of protein have previously been speculated to be a cause of hindered antibody penetration [39]. Schnell et al. mention that these issues could not be overcome by increasing antibody amounts or permeabilization agent and that the use of fixation and permeabilization methods in immunofluorescence microscopy are a potential cause of artefacts. Thus, immunostaining benefits from comparison with alternative visualization methods, such as fusion protein imaging in live-cell microscopy. In our case, the penetration of Hoechst stain would be explained by the significantly smaller size of the fluorophore. Accordingly, our results indicate that ring-shaped structures based on secondary stains should be cautiously interpreted.

**Fig 9:**
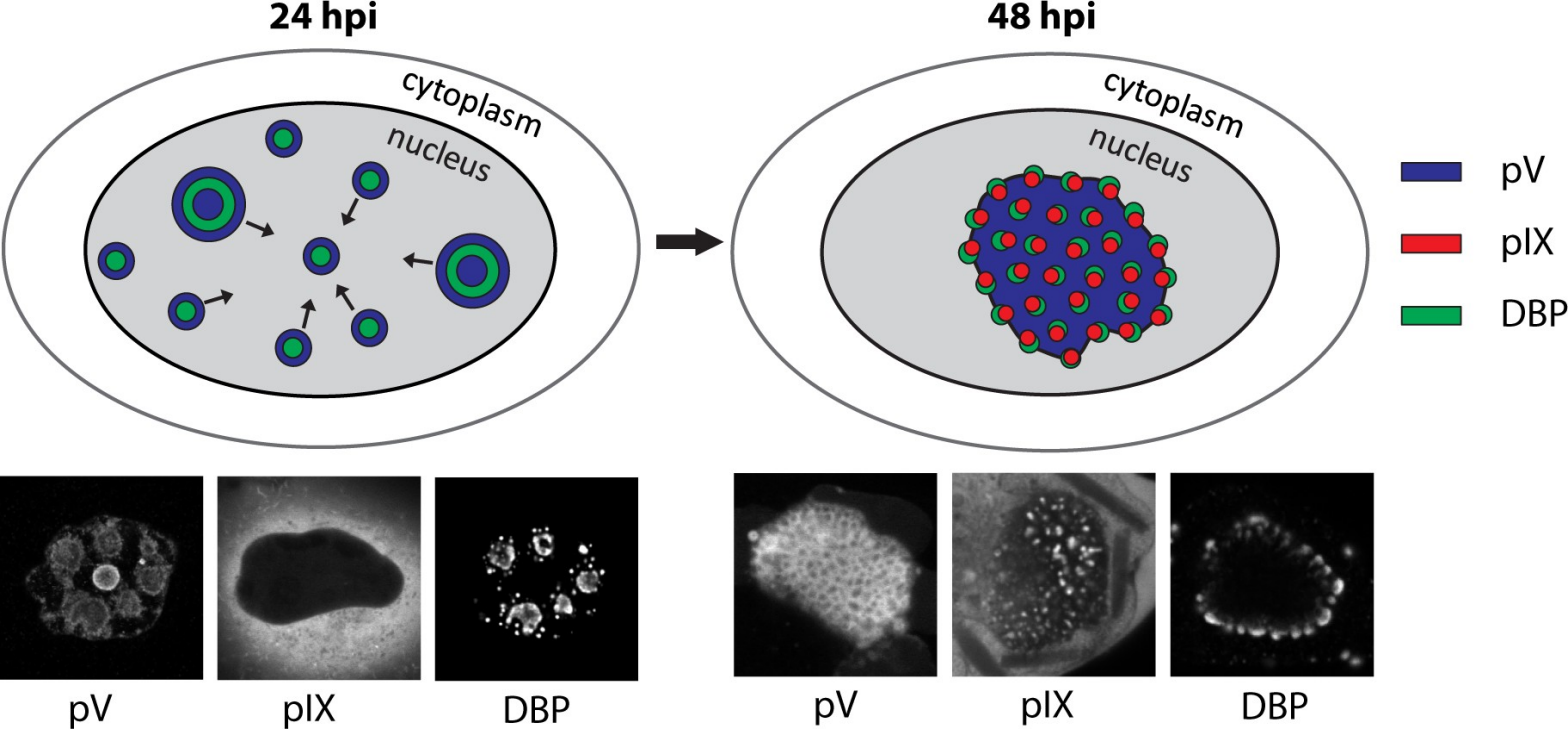
Model of late virion accumulation compartment (LVAC) formation and localization of viral proteins within. The nucleus is represented as a grey ellipse. At 24 hpi, pV is located around DBP replication centers in areas accumulating replicated genomes. At this timepoint, pIX was not found in the nucleus but was instead localized in the cytoplasm (not displayed in model). During transition between 24 and 48 hpi the viral proteins congregated to form the LVAC. Within the LVAC, pIX spots indicate the areas of accumulated assembled virus capsids in areas neighboring viral genomes and pV. Each protein localization is accompanied by a fluorescence microscopy image of the protein observed in our study.

This lack of antibody penetration should also be discussed for our DBP antibody stain at 48 hpi. In immunofluorescence staining we detected a ring formation of individual DBP spots localized around the LVAC (Fig 3). Similar individual DBP spots have been found by Genoveso et al. in late adenovirus replication compartments [40]. Such ring formation could be explained by two hypotheses. On the one hand, DBP replication centers could cluster on the outside of the LVAC and feed replicated viral genomes into the compartment. On the other hand, the LVAC could be filled with DBP replication centers of which only the outer layer is stained. Serving as an argument for the first hypothesis, a similar ring stain has been described for human cytomegalovirus (hCMV) DNA polymerase subunit UL44 [41]. Here, the use of a specific antibody resulted in a ring stain surrounding the hCMV replication compartment, which led to the hypothesis that replicated hCMV genomes are transported into the center of the replication compartment. However, a different UL44 antibody was shown to stain the protein throughout the replication compartment reducing the likeliness of this hypothesis [42]. In the second scenario, the DBP replication compartments are likely to occupy parts of the empty space within the pV honeycomb (Fig 9). In this case, DBP localization would resemble the localization of hCMV single-stranded DNA-binding protein UL55, which is spread throughout the replication compartment in multiple foci [43]. Supporting the latter scenario is our observation of a gradual signal decrease towards the center of the LVAC analogous to the pV and pIX staining results.

A logical way to test DBP localization within the LVAC would be to fluorescently tag DBP in the same manner as pV and pIX. However, mCherry fusion to DBP resulted in phenotypical artifacts compared to HAdV5 wt infection. In cells infected with DBP-mCherry no LVAC is formed and the DNA signal does not resemble a honeycomb-like organization (S5 Fig). Hence, even though fluorescent protein tags pose an effective toolset to further analyze viral protein localization in live cells, the possibility of introducing artifacts has to be carefully considered. Nonetheless, tagging pV and pIX has proven an effective strategy to retain virus replication whilst uncovering nuclear morphology changes during infection. Applying this toolset to other viral proteins is the logical next step.

### The LVAC formation is linked to DBP replication centers and retains assembled viruses as paracrystalline arrays

We hypothesize the involvement of the LVAC in HAdV5 assembly since the compartment contains replicated virus genomes and virus core and capsid proteins pV and pIX. Replication of viral genomes takes place within distinct nuclear replication centers containing DBP [7,9]. Komatsu et al postulated that in early replication centers (24 hpi) the replicated viral genomes spread to the surrounding and are histone-chromatinized for efficient transcription of late adenoviral genes. After an unknown switch mechanism, the replicated genomes accumulate in neighboring compartments titled virus-induced post-replication (ViPR) bodies, which are encircled by replicating genomes and DBP [32,33,40]. The nucleus of HAdV5 infected cells has been described to contain multiple ViPR bodies, which were shown to include chromatin remodeling proteins nucleophosmin-1 and Mybbp1a. Thus, ViPR bodies were implicated in adenoviral genome maintenance prior to packaging. Importantly, the ViPR bodies were demonstrated to be devoid of capsid proteins such as pVI and pIX [40]. In contrast, the LVAC is detectable a later timepoint in infection and was found to include pV and pIX, which are proteins incorporated into fully formed capsids. It is likely that the development of the LVAC is temporally positioned after ViPR body formation and that early replication centers develop first into late replication center including ViPR bodies and finally into the LVAC. This is aided by the observation that infected nuclei contain multiple late replication compartments and ViPR bodies whereas we detected a single large LVAC compartment later in infection.

Initially, pV is known to be excluded from early DBP replication foci and rings [18]. We also observed this exclusion at 24 hpi (Fig 2). Following the morphological changes of pV beyond this early time point, we detected the formation of ring-like intermediate pV-mCherry conformations before the protein fully formed the LVAC (Fig 5A). Both pV and Hoechst 33342 are known to bind double-stranded, but not single-stranded DNA. Therefore, it can be assumed that pV and Hoechst 33342 interact with the replicated viral genomes accumulating around DBP replication centers and not single-stranded replicating genomes within. Indeed, we observed a clear separation between DPB and Hoechst 33342 and pV signals. (Fig 5C).

During further redistribution of pV, the ring structures were lost and changed to the observed honeycomb organization (Fig 6). We quantified the colocalization of Hoechst 33342 and pV-mCherry in the compartment and showed that both occupy the same space. It can be assumed that within the LVAC both the Hoechst 33342 and pV-mCherry markers show the area of replicated viral genomes to be assembled to fully formed virions. An observation supporting our hypothesis of virus assembly within the LVAC is the localization of pIX-mCherry spots contiguous to pV. Ugai et al. observed the appearance of ‘nuclear speckles’ at a late time point in infection when tagging pIX with mRFP1 and EGFP, but they did not further elaborate on the formation or potential meaning of these speckles [37]. We could further link the pIX-mCherry spots with paracrystalline arrays of fully formed adenovirus capsids by combining fluorescence microscopy with transmission electron microscopy images of individual cells (Fig 7). Adenovirus as well as other non-enveloped viruses are known to form paracrystalline arrays [44–47]. The biological role of these virus arrays is not known. It is imaginable that newly formed virus capsids accumulate due to a lack of motility within the LVAC leading to tight virion clustering. Such clusters could then be dispersed upon cell rupture in infection. The apparent impermeability of the LVAC is shown through limited antibody penetration, but also by our FRAP and live-cell analysis of the pIX spots (Fig 8). In FRAP, only very little signal recovery occurred when bleaching pIX-mCherry. As the minor capsid protein pIX-mCherry is part of assembled virus capsids, an incorporation of the protein into virions would explain the observed immobilization. The pool of minor core protein pV-mCherry was only partially or weakly immobilized. An immobile fraction of 20% suggests that part of the protein is in tight association with DNA leaving 80% of pV-mCherry as a mobile protein phase which might in part prevent the penetration of antibodies and retain the virions within.

### Model of LVAC formation

Bringing our observations together, we propose a model of the nuclear morphology changes that occur during late stages of adenovirus infection (Fig 9). At 24 hpi, pV initially localizes throughout the nucleus and is interacting with the newly synthesized double-stranded viral genomes that are accumulating in the periphery of DBP replication centers and contributing to ViPR bodies. Those DPB rings and foci further congregate within the center of the nucleus. Following the path of DBP, pV also congregates within the center of the nucleus forming the LVAC at 48 hpi. Taken into account that the DBP stain does not penetrate the LVAC properly, we propose the LVAC to be filled by DBP foci surrounded by a phase of pV protein, which induces a honeycomb pattern of pV. The virions that are assembled in proximity to sites of viral replicated DNA and pV accumulate within the LVAC. As movement of virions within the LVAC is restricted, the newly-formed particles are spatially confined and therefore form paracrystalline virus arrays. These arrays subsequently appear as pIX-mCherry spots in fluorescence microscopy and show very limited signal recovery after photobleaching.

In summary, this study identified a late intranuclear compartment in which virus capsid and core proteins accumulate and virions can be detected as paracrystalline arrays. We showed that this compartment is not readily penetrable by antibodies, Furthermore, we proposed a model derived from the observations of protein and virus localization within the LVAC (Fig 9), which can be a useful tool to further study virion assembly at higher resolutions. It allows specific targeting of the LVAC for methods such as PALM/STORM microscopy or cryo-tomography after focused ion beam (FIB) milling.

## Methods

### Construction of HAdV5-mCherry fusion mutants

The adenoviral mutants were derived from the adenovirus strain H5*pg*4100 [48], lacking 1863 bp (nt 28602–30465) of the E3 region, with reinserted adenovirus death protein (ADP) which is termed wild-type (wt) in our study. The HAdV5 genome was modified and propagated in *Escherichia coli* within the low copy number plasmid p15A. Adenoviral mutants were generated via homologous recombination in a *ccdB* counterselection system [28]. Briefly, a *ccdB-amp* selection cassette was PCR amplified with primers carrying 50 bp homologous overhangs to the pV and pIX region of the adenovirus genome (Table 1). The *E*. *coli* strain GBred-*gyr*A462 was induced with 0.35% (w/v) L-arabinose and made electrocompetent by repeated washes with H_2_O. The p15A-HAdV5 vector together with the *ccdB-amp* PCR product were electroporated and cells were plated on LB agar plates (10 g/L tryptone, 5 g/L yeast extract, 5 g/L NaCl, 15 g/L agar) supplemented with 100 μg/mL ampicillin and 15 μg/mL chloramphenicol and grown at 37°C for 16 h. The DNA of appropriate clones was isolated by standard alkaline lysis and insertion of the cassette was verified by checking distinct restriction digestion patterns and Sanger sequencing. In a second recombination step, the *ccdb-amp* cassette was replaced with an mCherry-linker insert. Such constructs were synthesized (Eurofins Scientific) to be flanked by 400 bp homologous to the adenoviral *pV* or *pIX* locus on either side. The *E*. *coli* strain GB05-red was induced and made electrocompetent. The p15A-HAdV5-ccdb-amp vector together with the synthesized mCherry-linker insert was electroporated and cells were plated onto LB agar plates supplemented with 15 μg/mL chloramphenicol. The DNA of appropriate clones was isolated by standard alkaline lysis and insertion of the mCherry-linker region was verified by checking distinct restriction digestion patterns and Sanger sequencing. The linear viral genome was released from the p15A backbone by restriction digestion with PacI and SwaI enzymes.

**Table 1:** List of primers used for PCR amplification of *ccdB-amp* cassette.

pV ccdB amp fw	PCR	TAGACTCGTACTGTTGTATGTATCCAGCGGCGGCGGCGCGCAACGAAGCTGCCAGTATACACTCCGCTAG
pV ccdB amp rv	PCR	TCCGGCGCGATGACCTGGAGCATCTCTTCTTTGATTTTGCGCTTGGACATCAGCCCCATACGATATAAGTTG
pIX ccdB amp fw	PCR	TGCGCCAGCAGGTTTCTGCCCTGAAGGCTTCCTCCCCTCCCAATGCGGTTGCCAGTATACACTCCGCTAG
pIX ccdB amp rv	PCR	ACTTGCTTGATCCAAATCCAAACAGAGTCTGGTTTTTTATTTATGTTTTACAGCCCCATACGATATAAGTTG

### Mammalian cell line culture and generation of A549 lamin A mTagGFP cell line

Mammalian cell lines A549 (ATCC CCL-185), A549 lamin A mTagGFP, H1299 (ATCC CRL-5803) and MRC-5 (ATCC CCL-171) were cultured in Dulbecco’s Modified Eagle’s Medium (DMEM) (Gibco DMEM, high glucose, pyruvate, Thermo Scientific) with 1% (v/v) penicillin/streptomycin (P/S) solution (final 1000 U/ml penicillin and 1 mg/ml streptomycin, Pan-Biotech) and 10% (v/v) fetal calf serum (FCS) (FCS Superior, Merck). All cells were grown at 37°C and 5% (v/v) CO_2_.

A549 lamin A mTagGFP cells were generated by transduction of A549 cells with lentiviral particles encoding a lamin-chromobody (Chromotek). The lentiviral backbone has been described previously [49]. The lamin A chromobody open reading frame (orf) was inserted into the lentiviral backbone by replacing the BamHI/EcoRI fragment of the lentiviral backbone. The insert was previously amplified by PCR including an addition of MfeI and BglII restriction sites to the 3’ and 5’ end. Production of lentiviral particles and transduction procedure has been described previously [49]. In short, lentiviral particles were pseudotyped with VSV-g and produced in 293T cells. A549 cells were then transduced three times on three consecutive days. Afterwards, transduced cells were cultured in DMEM supplemented with 1% (v/v) penicillin/streptomycin (P/S) solution (final 1000 U/ml penicillin and 1 mg/ml streptomycin, Pan-Biotech) and 10% (v/v) fetal calf serum (FCS) (FCS Superior, Merck). Four days after the last transduction, cells were sorted for positive GFP signal (A549 lamin A mTagGFP poly). In order to generate a monoclonal cell line single cell-sorting was performed on A549 lamin A mTagGFP poly cells. Single cells were expanded and checked for GFP expression by flow-cytometry, which resulted in the monoclonal cell line A549 lamin A mTagGFP.

### Virus production, titration, infection and growth curve

New recombinant virus particles were produced in H1299 cells. A 10-cm culture dish of 90% confluent cells was transfected with lipofectamine 2000 and 1 μg linearized adenoviral genome. 7 days after transfection upon visible cytopathic effects virus particles were isolated by three freeze-thaw cycles. Cell debris was removed by centrifugation at 3400 g for 15 min and the supernatant was used to start a new round of infection for further passaging of the recombinant virus. After 5 passages the titer of the supernatant was determined via flow cytometry. A549 cells were seeded in 12 well plates and infected with a serial dilution of stock solution. After 24 h the cells were trypsinized, washed in PBS and fixed in 4% (v/v) PFA solution. The cells were resuspended in FACS buffer (1% (v/v) FCS in PBS) and analyzed on a LSRFortessa cell analyzer (BD Biosciences). The percentage of live, individual fluorescent cells was used to determine the fluorescence forming units (ffu) per μl of virus stock solution.

HAdV5 infection was performed at a multiplicity of infection (MOI) of 1 ffu/cell. For infection, the cells together with virus stock were incubated in DMEM (FCS and P/S free) for 1 h. Subsequently, DMEM with 1% (v/v) P/S and 10% (v/v) FCS was added in equal amounts leading to a final supplement concentration of 0.5% (v/v) P/S and 5% (v/v) FCS.

HAdV5 virus release growth curves were performed in A549 cells. 1.5×10^5^ cells were infected with a MOI of 1 in triplicate. The supernatant medium was carefully collected at 0, 24, 48, 72 and 96 hpi and stored at -80°C. 20 μl of supernatant was used to infect 2×10^5^ A549 cells. The cells were trypsinized, washed in PBS and fixed in 100% (v/v) methanol. The cells were blocked in FACS buffer (1% (v/v) FCS in PBS) and stained for DNA-binding protein using primary mouse B6-8 anti-DBP antibody [50]. Secondary antibody used was anti-mouse Alexa Fluor 488 (Invitrogen). The cells were resuspended in FACS buffer and analyzed on a LSRFortessa cell analyzer (BD Biosciences). The percentage of live, individual fluorescent cells was used to determine the fluorescence forming units (ffu) per μl of infection supernatant.

### Live-cell fluorescence microscopy

Appropriate cell lines were plated on culture dishes (μ-Dish 35 mm, glass bottom, ibidi) that were coated with 1% (v/v) fibronectin (Sigma Aldrich) for 40 min, infected with varying HAdV5 strains and prepared at 24 or 48 hpi for imaging of proteins of interest. Images were recorded on an inverted confocal spinning-disk microscope (Nikon Eclipse Ti-2 stand; Yokogawa CSU-W1 spinning disk; 2x Andor888 EM-CCD camera; Nikon 100x oil-immersion numerical aperture (NA) 1.49 objective) equipped with a heating chamber at 37°C and 5% (v/v) CO_2_. Images were recorded using the Nikon NIS-Elements software. Further image processing was performed in Fiji [51]. Colocalization quantification of fluorescence signal was performed using the Fiji JaCoP colocalization plugin [52]. In JaCoP, Mander’s colocalization coefficients were based on a background threshold, that is calculated by the plugin based on the input images to remove noise contribution within the image.

### Immunofluorescence microscopy

Appropriate cell lines were plated on culture dishes (μ-Dish 35 mm, glass bottom, ibidi) that were coated with 1% (v/v) fibronectin (Sigma Aldrich) for 40 min, infected with varying HAdV5 strains and prepared at 24 or 48 hpi for analysis of localization of proteins of interest. The cells were washed in PBS, fixed with 4% (v/v) paraformaldehyde (PFA) in PBS for 20 min and washed with PBS again. Afterwards, the cells were quenched with 25 mM NH_4_Cl in H_2_O for 10 min, washed in PBS and permeabilized with 0.5% (v/v) Triton X-100 for 10 min. After a PBS wash, cells were blocked with 1x TBS-BG solution (20 mM Tris/HCL pH 7.6, 137 mM NaCl, 3 mM KCl, 1.5 mM MgCl_2_, 0.05% (v/v) Tween 20, 5 mg/mL bovine serum albumin, 5 mg/mL glycine). After a PBS wash, the cells were stained for 1 h with primary antibodies, washed with PBS and stained for 30 min with secondary antibodies and Hoechst 33342 (0.05% (v/v). Primary antibodies used were mouse B6-8 anti-DBP [50], rabbit anti-pV and rabbit anti-pIX (kindly provided by Harald Wodrich (University of Bordeaux). Secondary antibodies used were anti-mouse Alexa Fluor 647 (Invitrogen), anti-rabbit Alexa Fluor 488 (Invitrogen), anti-rabbit Alexa Fluor 555 (Invitrogen). Images were recorded on an inverted confocal scanning laser microscope (Nikon A1R HD25 equipped with a Nikon 60x oil-immersion NA 1.40 objective). Images were recorded using the Nikon NIS-Elements software. Further image processing was performed in Fiji [51]. Colocalization quantification of fluorescence signal was performed using the Fiji JaCoP colocalization plugin [52]. Statistical significance was calculated using the unpaired Student’s t-test.

### Fluorescence recovery after photobleaching

A549 cells were plated on culture dishes (μ-Dish 35 mm, glass bottom, ibidi), infected with HAdV5 pIX-mCherry and HAdV5 pV-mCherry strains selected 48 hpi for FRAP analysis. Imaging was performed on an inverted confocal scanning laser microscope (Nikon A1R HD25 equipped with a Nikon 60x oil-immersion NA 1.40 objective). Stimulated image acquisition was performed using the Nikon NIS-Elements software. Three regions of interest (ROIs) of 1 μm diameter including the stimulation ROI (A), a reference ROI (R) and a background ROI (BG) were selected. The experimental sequence consisted of 10 sec of acquisition, a laser pulse at the stimulation ROI at 561 nm excitation and 120 sec of subsequent acquisition. Relative fluorescence intensities (I) were calculated according to following steps:

### Background correction of stimulation and reference ROIs

$$A_{corrected1}(t)=A(t)-BG(t)$$ (1)

$$R_{corrected1}(t)=R(t)-BG(t)$$ (2)

Reference correction of stimulation ROI

$$A_{corrected2}(t)=\frac{A_{corrected1}(t)}{R_{corrected1}(t)}$$ (3)

Normalization of stimulation ROI pre-bleach level

$$A_{corrected3}(t)=\frac{A_{corrected2}(t)}{A_{corrected2}(pre‐bleach)}$$ (4)

Mobile fractions were calculated by curve fitting the signal intensities after bleaching in Fiji. Assuming exponential recovery described by the formula:

$$y=a\times (1-e^{\left(-b\times x\right)})+c$$ (5)

with

*a* being a slowly recovering fraction

*c* being a rapidly diffusion fraction

*b* being the recovery rate

The fraction of mobile protein is given by:

$$f_{mobile}=a+c$$ (6)

### Comparative TEM and FM of cells: Sample preparation and microscopy

Appropriate cell lines were plated on culture dishes (μ-Dish 35 mm, high Grid-500, ibidi), infected with varying HAdV5 strains and prepared at 48 hpi for TEM analysis. The grid numbering on the cell culture dish allowed for identifying individual cells of interest throughout the sample preparation. For correlative analysis, the cells were imaged on an inverted confocal spinning-disk microscope (Nikon Eclipse Ti-2 stand; Yokogawa CSU-W1 spinning disk; 2x Andor888 EM-CCD camera; Nikon 100x oil-immersion numerical aperture (NA) 1.49 objective) equipped with a heating chamber at 37°C and 5% (v/v) CO_2_ directly before TEM preparation. Images were recorded using the Nikon NIS-Elements software. Further image processing was performed in Imaris (Oxford Instruments). Afterwards, the cells were washed with PBS and fixed with 2.5% (v/v) glutaraldehyde in PBS. After a PBS wash, the cells were stained with 1% (w/v) OsO_4_ in PBS for 20 min, washed with PBS followed by H_2_O and stained with 1% (w/v) uranyl acetate in H_2_O for 20 min. After a H_2_O wash, the cells were sequentially dehydrated in 50%, 70%, 90% and 100% (v/v) ethanol solution. Cells were embedded in epon resin [53]. Embedding was performed by incubation with 1:1 ethanol/epon mixture for 30 min, 3:7 ethanol/epon mixture overnight and 100% epon for 6 h. Polymerization was carried out at 60°C for 48 h. Ultrathin (~50 nm) sections were obtained on a microtome (Leica Ultracut EM UCT, Leica Microsystems, Austria), collected on carbon-coated copper grids (100 mesh, Plano), post-contrasted with saturated uranyl acetate solution in 70% (v/v) ethanol and imaged on a transmission electron microscope equipped with a LaB6 cathode operated at 80 kV (CM120 (Philips, Eindhoven, Netherlands; Multiscan 794 camera (Gatan, Pleasanton, CA, USA))

### TEM of purified virus: negative stain preparation and microscopy

Virus particles were purified on a single CsCl_2_ gradient (1.25 g/cm^3^/1.4 g/cm^3^) via ultracentrifugation at 104,000 g for 3 h at 18°C (Optima L-90K Ultracentrifuge, Beckman Coulter). Purified virus particles were fixed by addition of PFA to a final concentration of 4% (v/v). Virus particles were absorbed to 200 mesh copper grids (Plano) and negatively stained with 2% uranyl acetate. Particles were imaged with a transmission electron microscope equipped with a LaB6 cathode operated at 80 kV (CM120 (Philips, Eindhoven, Netherlands; Multiscan 794 camera (Gatan, Pleasanton, CA, USA)).

## Supporting information

S1 Fig A549 lamin A mTagGFP cell line allows to visualize the nuclear lamina. A549 cells expressing a GFP-nanobody construct recognizing lamin A were imaged by live-cell confocal laser-scanning fluorescence microscopy. A representative overview is shown. The nuclear lamina was stained by the GFP-nanobody (lamin A-TagGFP) and co-stained with a lamin A antibody (anti-lamin A). The signal overlap is represented in color (merge). The scalebar indicates 10 μm. Both signals colocalize with the difference that additional, free lamin A-nanobody was detected throughout the cells.
(TIF)

S2 Fig LVAC formation can be detected in H1299 cells. (A) Infection of H1299 cells with HAdV5 pV-mCherry at 24 hpi and 48 hpi. (B) Infection of H1299 cells with HAdV5 pIX-mCherry at 24 hpi and 48 hpi. The cells were imaged by live-cell confocal laser-scanning fluorescence microscopy. A representative cell is shown for each condition. The dsDNA signal is represented by Hoechst 33342 stain (Hoechst). The nuclear lamina is represented by a GFP-nanobody recognizing lamin A (Lamin A). pV and pIX localization is detected through the viral pV-mCherry and pIX-mCherry fusion construct (pV-mCherry/pIX-mCherry). The signal overlap is represented in color (merge). Scalebars indicate 10 μm.
(TIF)

S3 Fig LVAC formation can be detected in MRC-5 cells. (A) Infection of MRC-5 cells with HAdV5 pV-mCherry at 24 hpi and 48 hpi. (B) Infection of MRC-5 cells with HAdV5 pIX-mCherry at 24 hpi and 48 hpi. The cells were imaged by live-cell confocal laser-scanning fluorescence microscopy. A representative cell is shown for each condition. The dsDNA signal is represented by Hoechst 33342 stain (Hoechst). The nuclear lamina is represented by a GFP-nanobody recognizing lamin A (Lamin A). pV and pIX localization is detected through the viral pV-mCherry and pIX-mCherry fusion construct (pV-mCherry/pIX-mCherry). The signal overlap is represented in color (merge). Scalebars indicate 10 μm.
(TIF)

S4 Fig HAdV5 pV-mCherry and HAdV5 pIX-mCherry infection display a ring of DBP around LVAC at 48 hpi. (A) Immunofluorescence labeling of pV and DBP in HAdV5 pV-Cherry infection. (B) Immunofluorescence labelling of pIX and DBP in HAdV5 pIX-mCherry infection. A549 cells were infected with HAdV5 pV-Cherry/HAdV5 pIX-Cherry, fixed at 48 hpi, and imaged by confocal laser-scanning fluorescence microscopy. Cells were stained with Hoechst 33342 (Hoechst), and immunostained against pV (anti-pV) or pIX (anti-pIX) and DBP (anti-DBP). pV and pIX localization is detected through the viral pV-mCherry and pIX-mCherry fusion construct (pV-mCherry/pIX-mCherry). The signal overlap is represented in color (merge). A representative non-infected and infected cell is shown for each stain. Scalebars indicate 10 μm.
(TIF)

S5 Fig LVAC formation cannot be detected when infecting with a DBP-mCherry labelled virus mutant. The Infection of A549 cells with HAdV5 DBP-mCherry was analyzed at 24 hpi and 48 hpi. The cells were imaged by live-cell confocal spinning-disk fluorescence microscopy. A representative cell is shown for each condition. The dsDNA signal is represented by Hoechst 33342 stain (Hoechst). The nuclear lamina is represented by a GFP-nanobody recognizing lamin A (Lamin A). DBP localization is detected through the viral DBP-mCherry fusion construct (DBP-mCherry). The signal overlap is represented in color (merge). Scalebars indicate 10 μm.
(TIF)
