# Supplementary figures and images for: Fluorescent protein tagging of adenoviral proteins pV and pIX reveals ‘late virion accumulation compartment’
Source: PLoS Pathog. 2020 Jun 25;16(6):e1008588. doi: 10.1371/journal.ppat.1008588 (PMC7343190; doi:10.1371/journal.ppat.1008588)

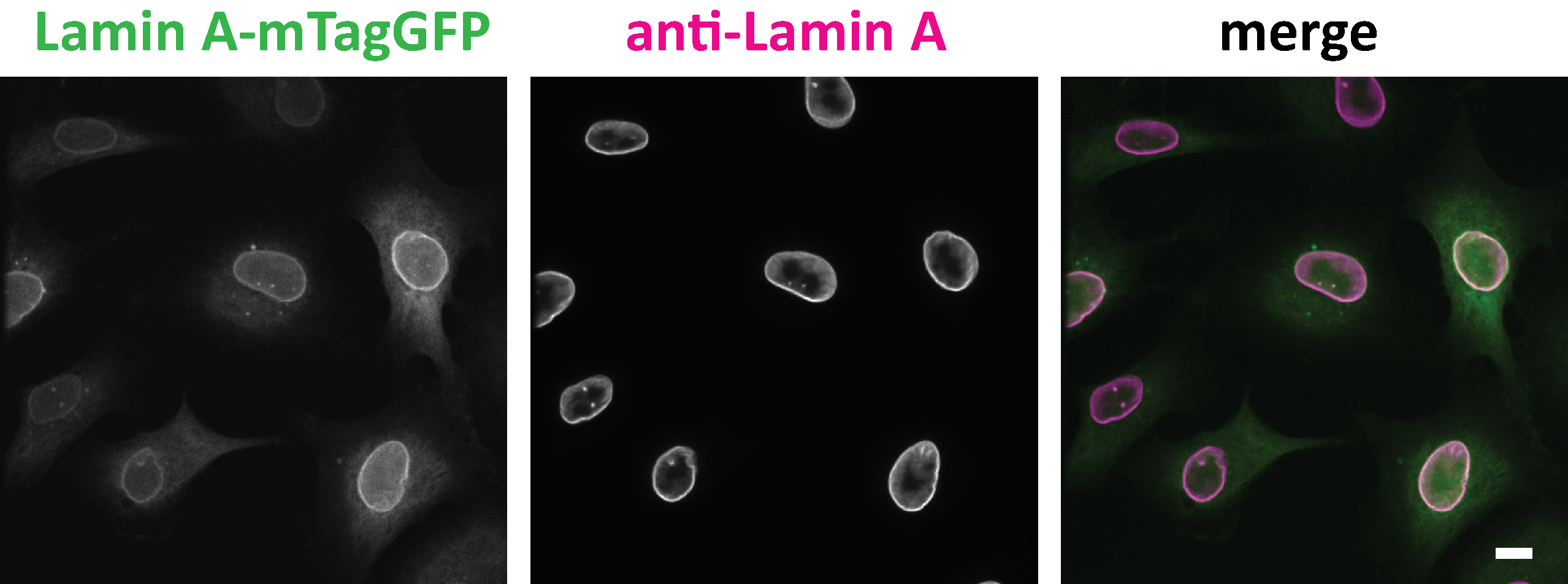

Supplement: S1 Fig — A549 cells expressing a GFP-nanobody construct recognizing lamin A were imaged by live-cell confocal laser-scanning fluorescence microscopy. A representative overview is shown. The nuclear lamina was stained by the GFP-nanobody (lamin A-TagGFP) and co-stained with a lamin A antibody (anti-lamin A). The signal overlap is represented in color (merge). The scalebar indicates 10 μm. Both signals colocalize with the difference that additional, free lamin A-nanobody was detected throughout the cells. (TIF) [file ppat.1008588.s001.tif]

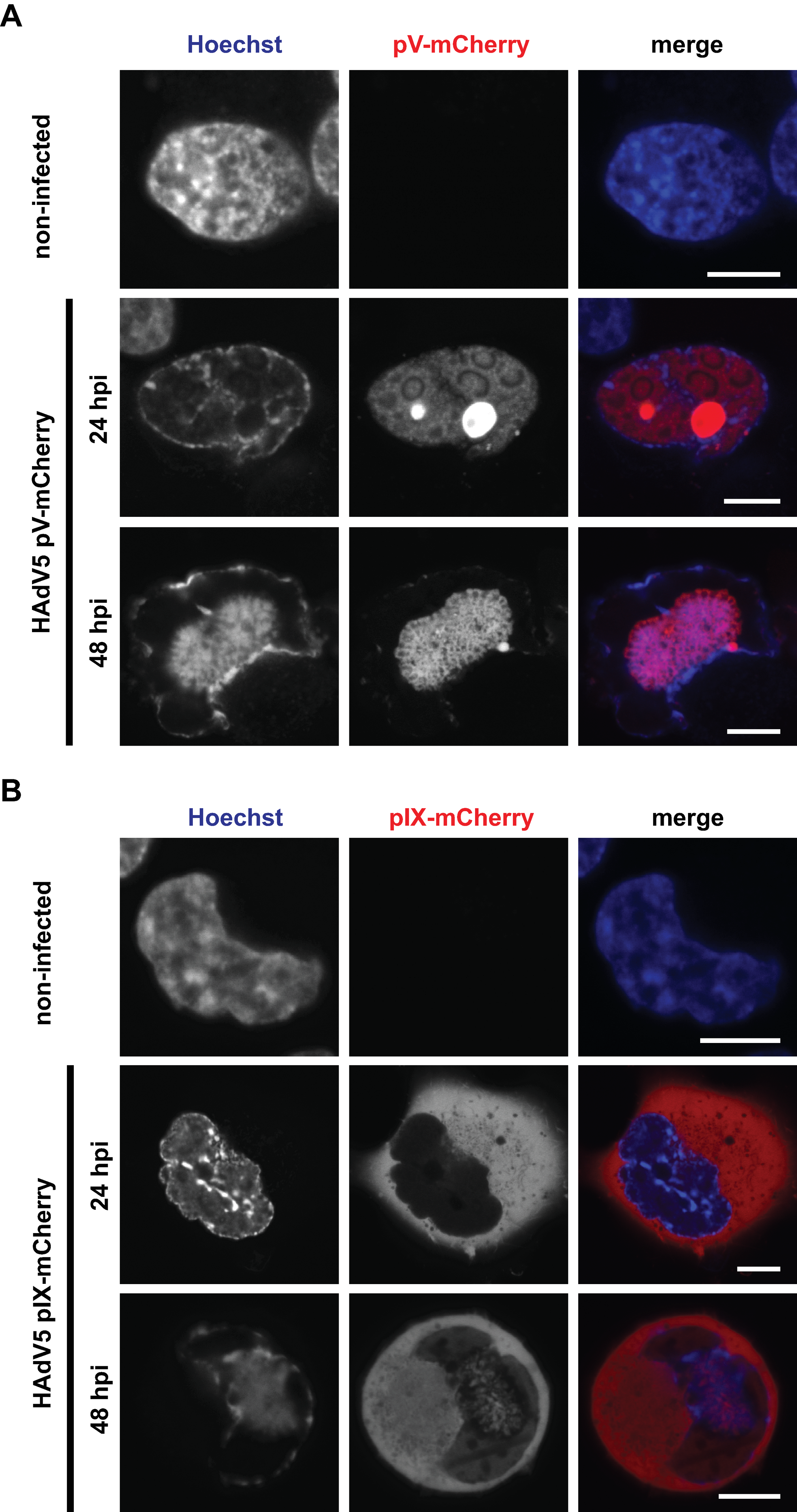

Supplement: S2 Fig — (A) Infection of H1299 cells with HAdV5 pV-mCherry at 24 hpi and 48 hpi. (B) Infection of H1299 cells with HAdV5 pIX-mCherry at 24 hpi and 48 hpi. The cells were imaged by live-cell confocal laser-scanning fluorescence microscopy. A representative cell is shown for each condition. The dsDNA signal is represented by Hoechst 33342 stain (Hoechst). The nuclear lamina is represented by a GFP-nanobody recognizing lamin A (Lamin A). pV and pIX localization is detected through the viral pV-mCherry and pIX-mCherry fusion construct (pV-mCherry/pIX-mCherry). The signal overlap is represented in color (merge). Scalebars indicate 10 μm. (TIF) [file ppat.1008588.s002.tif]

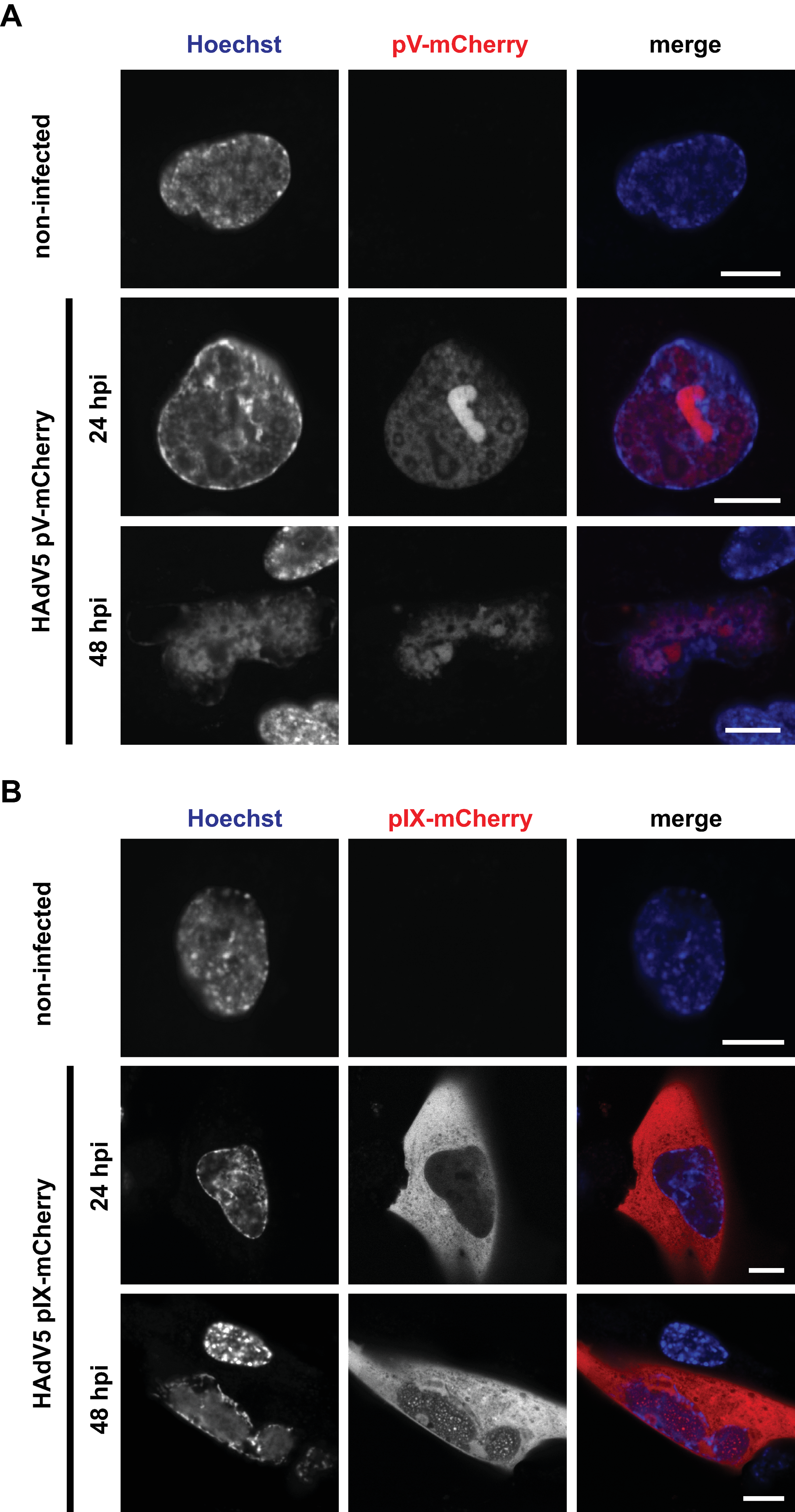

Supplement: S3 Fig — (A) Infection of MRC-5 cells with HAdV5 pV-mCherry at 24 hpi and 48 hpi. (B) Infection of MRC-5 cells with HAdV5 pIX-mCherry at 24 hpi and 48 hpi. The cells were imaged by live-cell confocal laser-scanning fluorescence microscopy. A representative cell is shown for each condition. The dsDNA signal is represented by Hoechst 33342 stain (Hoechst). The nuclear lamina is represented by a GFP-nanobody recognizing lamin A (Lamin A). pV and pIX localization is detected through the viral pV-mCherry and pIX-mCherry fusion construct (pV-mCherry/pIX-mCherry). The signal overlap is represented in color (merge). Scalebars indicate 10 μm. (TIF) [file ppat.1008588.s003.tif]

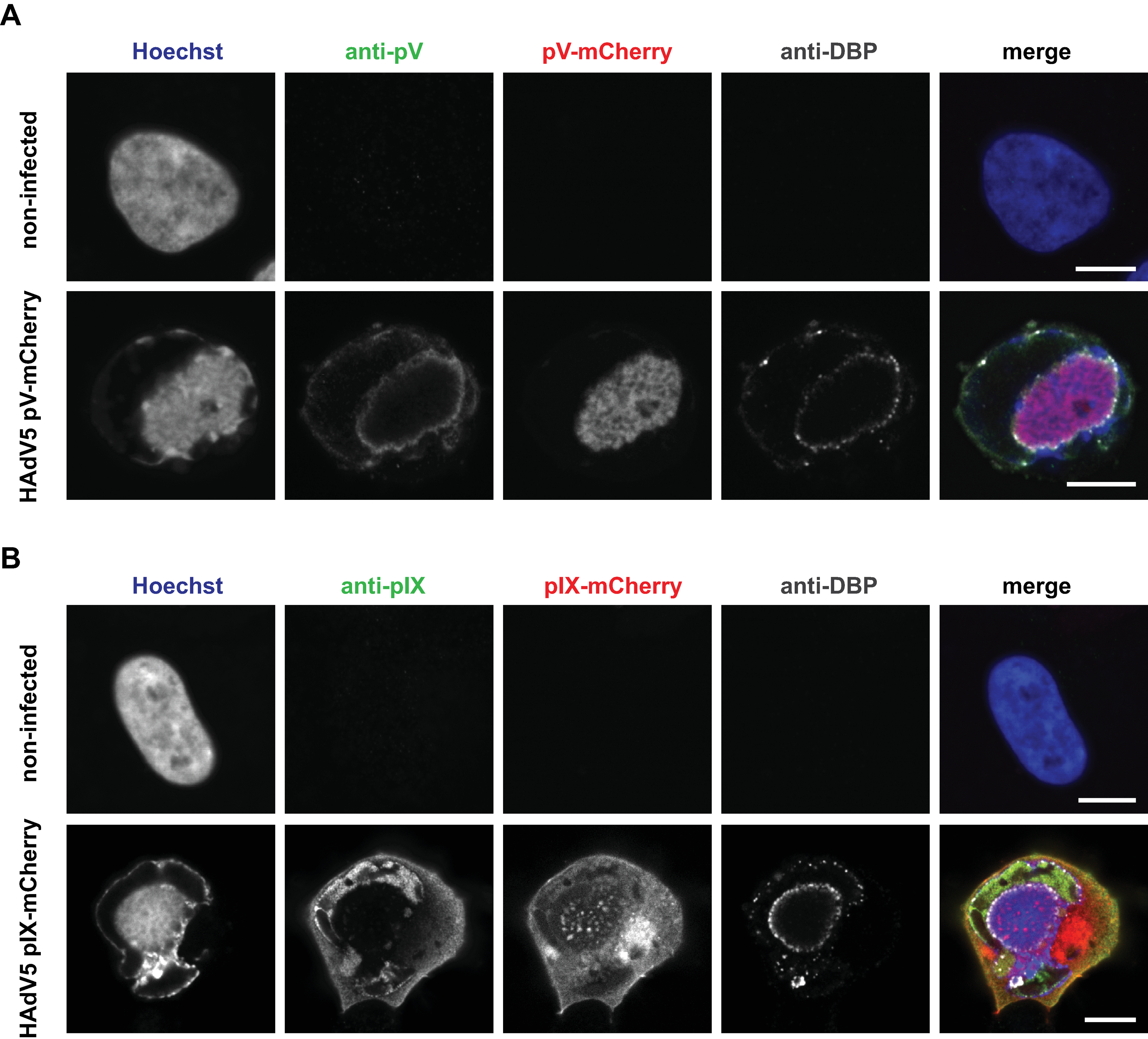

Supplement: S4 Fig — (A) Immunofluorescence labeling of pV and DBP in HAdV5 pV-Cherry infection. (B) Immunofluorescence labelling of pIX and DBP in HAdV5 pIX-mCherry infection. A549 cells were infected with HAdV5 pV-Cherry/HAdV5 pIX-Cherry, fixed at 48 hpi, and imaged by confocal laser-scanning fluorescence microscopy. Cells were stained with Hoechst 33342 (Hoechst), and immunostained against pV (anti-pV) or pIX (anti-pIX) and DBP (anti-DBP). pV and pIX localization is detected through the viral pV-mCherry and pIX-mCherry fusion construct (pV-mCherry/pIX-mCherry). The signal overlap is represented in color (merge). A representative non-infected and infected cell is shown for each stain. Scalebars indicate 10 μm. (TIF) [file ppat.1008588.s004.tif]

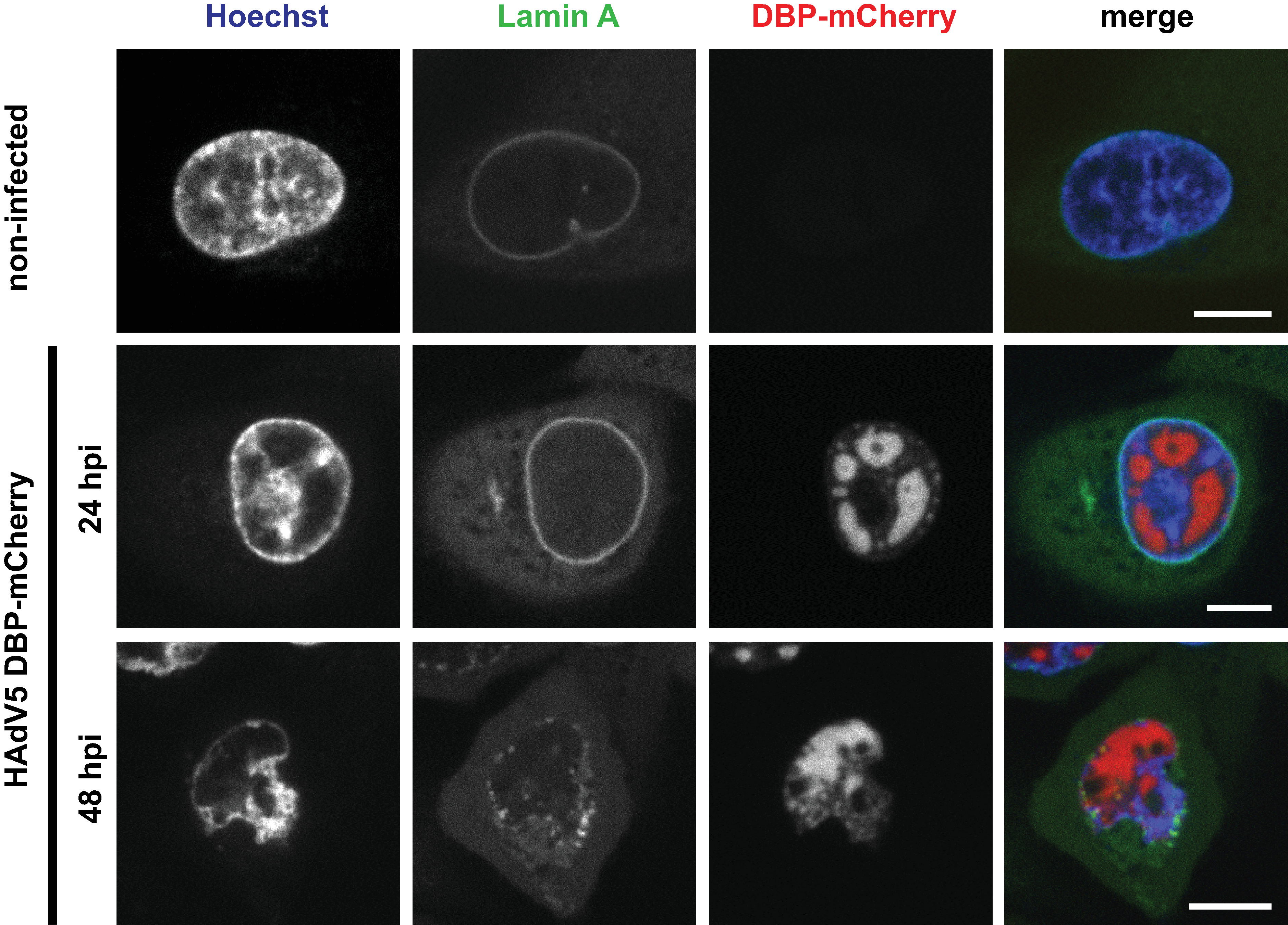

Supplement: S5 Fig — The Infection of A549 cells with HAdV5 DBP-mCherry was analyzed at 24 hpi and 48 hpi. The cells were imaged by live-cell confocal spinning-disk fluorescence microscopy. A representative cell is shown for each condition. The dsDNA signal is represented by Hoechst 33342 stain (Hoechst). The nuclear lamina is represented by a GFP-nanobody recognizing lamin A (Lamin A). DBP localization is detected through the viral DBP-mCherry fusion construct (DBP-mCherry). The signal overlap is represented in color (merge). Scalebars indicate 10 μm. (TIF) [file ppat.1008588.s005.tif]
